# Pathogen prioritisation for wastewater surveillance ahead of the Paris 2024 Olympic and Paralympic Games, France

**DOI:** 10.2807/1560-7917.ES.2024.29.28.2400231

**Published:** 2024-07-11

**Authors:** Laila Toro, Henriette de Valk, Laura Zanetti, Caroline Huot, Arnaud Tarantola, Nelly Fournet, Laurent Moulin, Ali Atoui, Benoît Gassilloud, Damien Mouly, Frédéric Jourdain

**Affiliations:** 1 Santé publique France (French National Public Health Agency), Montpellier, France; 2 Santé publique France (French National Public Health Agency), Saint-Maurice, France; 3 Institut national de santé publique du Québec, Québec, Canada; 4 Santé publique France (French National Public Health Agency), Saint-Denis, France; 5 Eau de Paris, Ivry-sur-Seine, France; 6 ANSES Nancy Laboratory for Hydrology, Nancy, France; 7 Santé publique France (French National Public Health Agency), Toulouse, France

**Keywords:** wastewater, surveillance, mass gathering, Olympic, Paralympic, Delphi, pathogen, public health

## Abstract

**Background:**

Wastewater surveillance is an effective approach to monitor population health, as exemplified by its role throughout the COVID-19 pandemic.

**Aim:**

This study explores the possibility of extending wastewater surveillance to the Paris 2024 Olympic and Paralympic Games, focusing on identifying priority pathogen targets that are relevant and feasible to monitor in wastewater for these events.

**Methods:**

A list of 60 pathogens of interest for general public health surveillance for the Games was compiled. Each pathogen was evaluated against three inclusion criteria: (A) analytical feasibility; (B) relevance, i.e. with regards to the specificities of the event and the characteristics of the pathogen; and (C) added value to inform public health decision-making. Analytical feasibility was assessed through evidence from peer-reviewed publications demonstrating the detectability of pathogens in sewage, refining the initial list to 25 pathogens. Criteria B and C were evaluated via expert opinion using the Delphi method. The panel consisting of some 30 experts proposed five additional pathogens meeting criterion A, totalling 30 pathogens assessed throughout the three-round iterative questionnaire. Pathogens failing to reach 70% group consensus threshold underwent further deliberation by a subgroup of experts.

**Results:**

Six priority targets suitable for wastewater surveillance during the Games were successfully identified: poliovirus, influenza A virus, influenza B virus, mpox virus, SARS-CoV-2 and measles virus.

**Conclusion:**

This study introduced a model framework for identifying context-specific wastewater surveillance targets for a mass gathering. Successful implementation of a wastewater surveillance plan for Paris 2024 could incentivise similar monitoring efforts for other mass gatherings globally.

**Key public health message**

**What did you want to address in this study and why?**

The growing body of evidence on the effective use of wastewater surveillance (WWS) to monitor population health highlights its potential for broader application. We explored the possibility of extending WWS to the Paris 2024 Olympic and Paralympic Games. Our aim was to identify relevant and feasible pathogen targets for WWS during these mass gathering events while using a reproducible, transparent and qualitative framework.

**What have we learnt from this study?**

Using evidence from peer-reviewed publications and expert opinion, six priority pathogen targets suitable for WWS during the Paris 2024 Olympic and Paralympic Games were successfully identified: poliovirus, influenza A virus, influenza B virus, mpox virus, SARS-CoV-2 and measles virus.

**What are the implications of your findings for public health?**

This study introduced a model framework for identifying context-specific WWS targets for mass gatherings and offered a starting point for WWS planning for the Paris 2024 Olympic and Paralympic Games. If successful, WWS for the Games and the lessons learned from its design and implementation could serve as an incentive for the adoption of WWS during other mass gathering events worldwide.

## Introduction

Wastewater acts as a ‘mirror’ of population health. Through the analysis of sewage samples, wastewater-based epidemiology (WBE) provides information on substance consumption (licit and illicit drugs, tobacco and alcohol), chemical exposure and the circulation of certain pathogens within a population [1,2]. While it gained considerable momentum during the COVID-19 pandemic, wastewater surveillance (WWS) is not a new concept. The tool has been used to follow waterborne or faecal-oral transmitted pathogens such as the poliovirus for several decades [1,3]. Nonetheless, since it was proven in 2020 that the genome of severe acute respiratory syndrome coronavirus 2 (SARS-CoV-2) could be detected in wastewater and that its quantification correlated with COVID-19 cases, the field of WBE and its methods have developed considerably [1,4]. The environmental surveillance of SARS-CoV-2 offers many advantages, as it: (i) collects data regardless of health status (symptomatic, asymptomatic); (ii) is not influenced by the availability of testing or test-seeking behaviour; (iii) can act as an early warning system; (iv) is cost-effective and (v) provides community-level data in a passive and non-intrusive manner [1,2,4]. Further, with the progression of the COVID-19 pandemic and emergence of SARS-CoV-2 variants of concern, WWS objectives have expanded beyond the mere detection of virus presence and monitoring of trends to include virus quantification and the assessment of genetic diversity. Indeed, wastewater data has been used extensively to complement other population-based surveillance systems and can help guide public health decision-making [1,3-5]. The success of WBE over the last few years has raised questions about the possibilities of extending this surveillance to other infectious agents and contexts. One such example is the public health surveillance of mass gathering (MG) events.

The World Health Organization (WHO) characterises an MG as an event where the volume of attendees can stretch the planning and response capabilities of the community, state or nation hosting the event [6]. Such gatherings have inherent characteristics that place them at greater risk of infectious disease transmission. Mass gathering events involve the concentration of large numbers of people in the same place at the same time, which facilitates pathogen dissemination. These gatherings may also see a considerable influx of international travellers, increasing the risk of disease importation [6,7]. Travellers may also exhibit modified individual health seeking behaviours because of cost, language barriers and/or lack of familiarity with the healthcare system on location. Although rare, notable examples of epidemics related to MGs have been described in the literature [6,8-11]. Moreover, an epidemic occurring during an international MG has a greater potential to be exported from the host country, and therefore of becoming a cross-border health issue [6]. From 26 July to 8 September 2024, France will host the Olympic and Paralympic Games (OPG), the two largest international sporting MG events. The Paris 2024 OPG are expected to bring over 15.9 million visitors cumulatively and will be the largest sporting events held in the country to date [12,13]. In this context, France’s public health preparedness is being adapted, notably by reinforcing health monitoring and epidemiological surveillance. The inclusion of WWS as a component of the enhanced surveillance system under development for the Paris Games was suggested.

This study sought to identify priority pathogen targets that are both relevant and feasible to monitor in wastewater during the Paris 2024 Games, while using a reproducible, transparent and qualitative framework. It constitutes the first step taken towards the conception and planning of a WWS strategy for Paris 2024.

## Methods

### Identification of pathogens/diseases for public health surveillance

To identify pathogens/diseases of interest for general public health surveillance (non-wastewater-specific) for the 2024 OPG, a preliminary list of pathogens/diseases of relevance for the surveillance of MGs was compiled by the authors of this manuscript using the following criteria (Box).

Box **Criteria for identifying pathogens/diseases of interest for general public health surveillance during the Paris 2024 Olympic and Paralympic Games, France**

- pathogens and diseases identified by ECDC as surveillance priorities for the London 2012 and Rio de Janeiro 2016 OPG [7,40].
- infectious diseases identified as requiring increased surveillance for the Tokyo 2020 OPG following risk assessments performed by the 47 districts in Japan hosting Olympic training centres and sporting events [41].
- infectious diseases that have caused epidemics at previous OPGs, from the 2002 Winter OPG in Salt Lake City to the 2020 Summer OPG in Tokyo [7,37,38].
- infectious diseases identified by the WHO as presenting the greatest risk to public health because of their epidemic potential and/or the absence or insufficiency of countermeasures [39].

ECDC: European Centre for Disease Prevention and Control; OPG: Olympic and Paralympic Games; WHO: World Health Organization.

The pathogens/diseases listed were then associated to disease categories identified as priorities for the Games by France’s national public health agency (Santé publique France, SpF) in a risk map created for the event. These categories included notifiable diseases in France, pathogens associated with food poisoning and/or food-borne illness outbreaks, acute respiratory infections, emerging infectious diseases and zoonoses. Only pathogens/diseases from the preliminary list that fit into these groups were retained. These, along with six additional infectious diseases identified specifically by SpF (brucellosis, West Nile fever, mpox, Venezuelan equine encephalitis, glanders and echinococcosis), were compiled into a list of pathogens to be considered for WWS during the Paris 2024 OPG.

### Framework for identifying priority pathogen targets for wastewater surveillance

**Figure 1:**
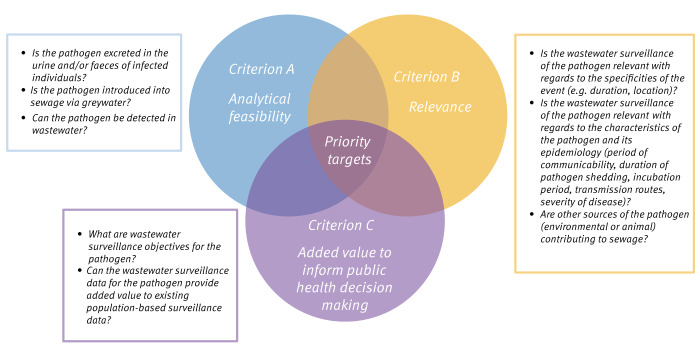
Framework for identifying priority targets for wastewater surveillance among pathogens deemed of interest for general public health surveillance during the Paris 2024 Olympic and Paralympic Games, France

Not all pathogens are adapted and relevant targets for WWS, especially during an MG event like the Paris 2024 OPG. To identify priority targets in this context, candidate pathogens identified in the previous step were evaluated against three inclusion criteria: (A) analytical feasibility; (B) relevance, i.e. with regards to the specificities of the event and the characteristics of the pathogen; and (C) added value to inform public health decision-making (Figure 1). This methodology was adapted from similar approaches used to prioritise pathogens for WWS in other contexts [4,14].

The figure is adapted from [4].

To be monitored in wastewater, pathogens must be excreted in the urine and/or faeces of infected individuals or introduced into sewage via greywater, i.e. from showers and other hygiene activities. Pathogens must also persist in the environment long enough to be detectable by existing analytical methods [4,15,16]. We considered analytical feasibility (criterion A) to be met if there was scientific evidence supported by at least one peer-reviewed publication that a pathogen has been detected in wastewater previously. The articles in question are provided in Supplementary Table S1, a table of key information on potential pathogens. This first criterion was evaluated via a literature search conducted using the PubMed database in March 2023. An example of the search query used is available in Supplement S1. Papers referencing spiked samples, open sewer systems or that sampled hospital effluents exclusively were excluded to better reflect the conditions that will be observed during the Paris 2024 Games.

Event- and pathogen-specific (and associated disease-specific) characteristics influence the relevance of implementing WWS for a pathogen. Further, to be retained, wastewater monitoring must add value to public health decision-making with regards to population-based surveillance already in place, i.e. nationally notifiable diseases, sentinel surveillance, emergency department admission rates, screening tests data, etc. To evaluate inclusion criteria B and C and establish a list of priority targets for WWS for the 2024 OPG, expert opinion was solicited using the Delphi method.

### Delphi method

A Delphi survey is a method for the systematic solicitation of opinions from a panel of experts on a particular topic. It aims to achieve consensus through a structured, anonymous process involving an iterative series of questionnaires. For our study, after each round, the questionnaire was adapted and pathogens for which consensus was reached were removed. Panellists also received the aggregated responses of respondents from the previous round and were given the opportunity to re-evaluate their own answers on this basis, revising them when deemed appropriate [17].

Experts were selected based on their professional background and complementary expertise in the fields of public health (with a focus on infectious diseases), WBE, microbiology and drinking-water/wastewater treatment. They were recruited from eight different organisations at the regional and national level in France, as well as one from Canada, and invited to participate via email. To mitigate potential bias related to the different states of knowledge of panellists on potential surveillance targets and to help inform pathogen evaluation, a summary of key information relating to pathogens that satisfied the analytical feasibility criterion, was prepared (provided as Supplementary Table S1). Only those experts having completed a round of the survey were invited to participate in its subsequent iteration.

Panellists were asked to judge whether pathogens would constitute suitable targets for wastewater monitoring during the Paris 2024 OPG. In line with previous studies [17], we considered group consensus reached when there was a convergence in expert opinion ≥ 70% with regards to a pathogen’s inclusion or exclusion from this surveillance. To be judged favourably, a participant needed to be convinced that the pathogen in question satisfied all three inclusion criteria listed above. When deemed suitable, experts were questioned on the pathogen’s most pertinent WWS objective(s), among: (i) detection (presence/absence); (ii) quantification; (iii) trend monitoring and (iv) assessment of genetic diversity. Alternatively, for pathogens they suggested to exclude from this surveillance, panellists were asked to justify their decision. At this stage, the relevance criterion was subcategorised into ‘relevance with regard to the specificities of the event’ and ‘relevance with regard to pathogen-/disease-specific characteristics’. The Delphi method took place over a nearly 3-month period from 3 May to 25 July 2023.

Pathogens failing to meet criterion A (i.e. analytical feasibility) because of a lack of evidence of detectability in wastewater were assembled in a catalogue of prospective pathogens for wastewater research. Participants were then tasked with identifying which among these should constitute priority research targets for the development of detection/quantification methods. Finally, experts were questioned about the need to consider other pathogens for this surveillance. An example of the questions asked for each infectious agent evaluated is available in Supplement S2.

The third round was set as the end of the iterative process, which corresponds to the standard duration of a Delphi process [17,18]. All data collected were treated anonymously. The electronic questionnaire was created using LimeSurvey (version 5.6.54), and auto-administrated through an active link.

### Assessment of pathogens below the consensus threshold

Pathogens failing to reach the 70% consensus threshold at the end of the Delphi exercise, were reassessed collaboratively by a group of SpF epidemiologists in charge of France’s national wastewater monitoring system (SUM’Eau). The following elements were considered: (i) proximity to the threshold for inclusion/exclusion; (ii) the pathogens’ epidemiological, clinical and microbiological characteristics, as well as (iii) comments left by panellists.

## Results

**Figure 2:**
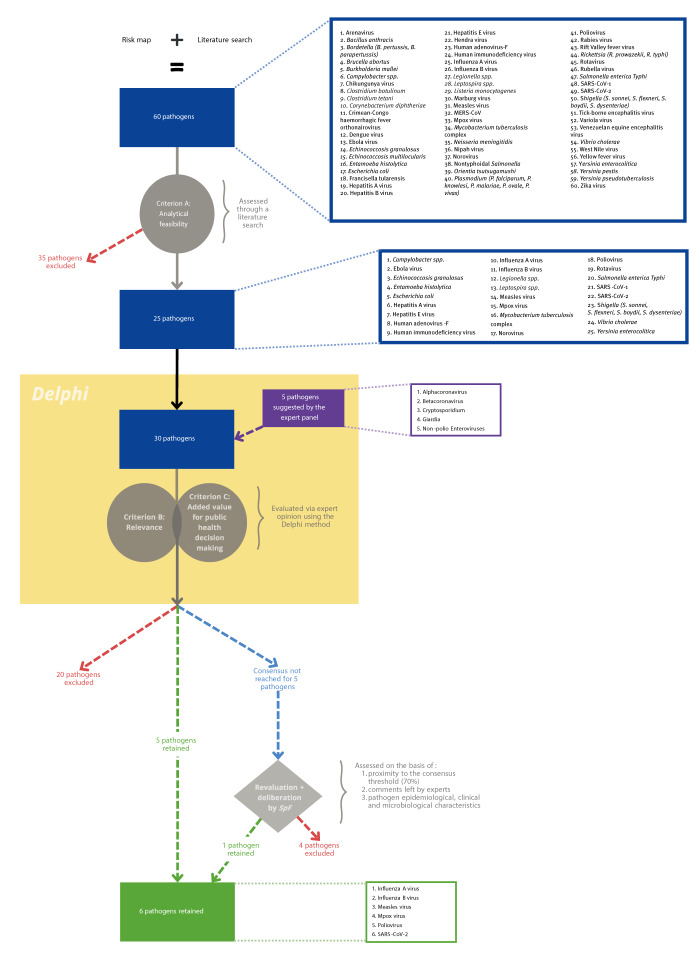
Flowchart summarising steps taken to identify priority targets for wastewater surveillance during the Paris 2024 Olympic and Paralympic Games, France

A list of pathogens/diseases of interest for public health surveillance for the Paris 2024 OPG was produced (Table 1) and included 60 causative agents, the details of which are provided in Supplementary Table S1. Evaluation based on criterion A (i.e. analytical feasibility) reduced this list to 25 pathogens (Figure 2).

**Table 1:** Pathogens/diseases of interest for public health surveillance during the Paris 2024 Olympic and Paralympic Games, France

Pathogen/disease	Communicable diseases deemed of relevance for increased surveillance activities following risk assessments			Infectious diseases occurrences in previous OPGs^a^ [7,37,38]	Diseases posing the greatest public health risk (WHO) [39]	Disease categories of interest identified by Santé publique France					Pathogens/ diseases of interest for public health surveillance during the Paris 2024 OPG
	London 2012 OPG [40]	Rio de Janeiro 2016 OPG [7]	Tokyo 2020 OPG [41]			Notifiable diseases in France	Pathogens responsible for food poisoning/food-borne illness outbreaks	Acute respiratory infections	Emerging infectious diseases	Zoonoses	
**Vaccine-preventable diseases**											
Diphtheria	1	-	-	-	-	1	**-**	**-**	**-**	**-**	1
Measles	1	1	1	1	-	1	**-**	**-**	**-**	**-**	1
Meningococcal disease	1	1	1	-	-	1	**-**	**-**	**-**	**-**	1
Mumps	-	1	-	-	-	**-**	**-**	**-**	**-**	**-**	**-**
Pertussis	1	1	1	-	-	**-**	**-**	1	**-**	**-**	1
Pneumococcal disease	1	-	-	-	-	**-**	**-**	**-**	**-**	**-**	**-**
Poliomyelitis	-	1	-	-	-	1	**-**	**-**	**-**	**-**	1
Rubella	-	1	1	-	-	1	**-**	**-**	**-**	**-**	1
Typhoid fever (Typhoidal *Salmonella*)	1	1	-	-	-	1	**-**	**-**	**-**	**-**	1
**Food- and waterborne diseases**											
Amoebiasis	-	1	-	-	-	-	1	-	-	-	1
Botulism	-	1	-	-	-	1	1	-	-	-	1
**Brucellosis**	-	-	-	-	-	1	1	-	-	1	1
*Campylobacter* infection	1	1	1	-	-	**-**	1	-	-	1	1
Cholera	1	-	-	-	-	1	1	-	-	-	1
*Escherichia coli* infection	1	1	1	-	-	**-**	1	-	-	1	1
Hepatitis A	1	1	1	-	-	1	1	-	-	-	1
Hepatitis E	-	-	1	-	-	-	1	-	-	1	1
Human adenovirus-F	1	-	-	-	-	-	1	-	-	-	1
Legionnaires’ disease	1	1	-	-	-	1	1	-	-	-	1
Norovirus	1	1	1	1	-	-	1	-	-	-	1
Rotavirus	1	-	-	-	-	-	1	-	-	-	1
Salmonellosis (nontyphoidal *Salmonella*)	1	1	1	-	-	-	1	-	-	1	1
Shigellosis	1	1	1	-	-	-	1	-	-	-	1
Yersiniosis	-	1	-	-	-	-	1	-	-	1	1
Food poisoning/ gastrointestinal illness (unspecified)	-	1	1	1	-	-	-	-	-	-	-
**Airborne diseases**											
Influenza	1	-	1	1	-	-	-	1	-	1	1
MERS-CoV	-	-	1	-	1	-	-	1	-	1	1
Tuberculosis	1	1	1	-	-	1	-	1	-	1	1
Respiratory tract infection (unspecified or other)	1	-	-	1	-	-	-	-	-	-	-
**Emerging and vector-borne diseases**											
Arenavirus diseases (Lassa, Junin, Machupo, Guanarito, and Sabiá fever)	1	-	-	-	1^b^	1	-	-	1	1	1
Chikungunya	-	1	1	-	-	1	-	-	1	1^c^	1
COVID-19	-	-	-	1	1	1	-	1	1	-	1
Crimean-Congo haemorrhagic fever	-	-	-	-	1	1	-	-	1	1	1
Dengue	-	1	1	-	-	1	-	-	-	1^c^	1
Ebola or Marburg viruses (filoviruses)	1	-	-	-	1	1	-	-	1	1	1
Invasive group A streptococcal infection	1	-	-	-	-	-	-	-	-	-	-
Malaria	-	-	-	-	-	1	-	-	-	1	1
**Mpox**	-	-	-	-	-	**-**	**-**	**-**	1	1	1
Rift Valley fever	-	-	-	-	1	1	-	-	1	1	1
Severe acute respiratory syndrome (SARS)	1	-	-	-	-	**-**	-	1	1	-	1
Smallpox	1	-	-	-	-	1	-	-	-	-	1
Tularaemia	-	-	-	-	-	1	-	-	-	1	1
Typhus	-	-	-	-	-	1	-	-	-	1	1
**West Nile virus infection**	-	-	-	-	-	1	-	-	-	1	1
Yellow fever	-	1	-	-	-	1	-	-	-	1	1
Zika virus disease	-	1	1	-	1	1	-	-	1	1^c^	1
**Zoonoses**											
Anthrax	1	-	-	-	-	1	-	-	-	1	1
**Echinococcosis**	-	-	-	-	-	**-**	**-**	**-**	**-**	1	1
**Glanders**	-	-	-	-	-	**-**	**-**	**-**	**-**	1	1
Leptospirosis	1	1	-	-	-	1	-	-	-	1	1
Nipah and henipaviral diseases	-	-	-	-	1	**-**	**-**	**-**	1	1	1
Rabies	1	-	-	-	-	1	-	-	-	1	1
**Venezuelan equine encephalitis**	-	-	-	-	-	**-**	**-**	**-**	**-**	1	1
**Sexually transmitted infections**											
Chlamydia infection	-	-	-	1	-	**-**	**-**	**-**	**-**	**-**	**-**
Hepatitis B	-	1	-	-	-	1	-	-	-	-	1
Hepatitis C	-	1	-	-	-	**-**	**-**	**-**	**-**	**-**	**-**
Human immunodeficiency virus infection	1	-	1	-	-	1	-	-	-	-	1
Syphilis	1	-	1	-	-	**-**	**-**	**-**	**-**	**-**	** -**
Genital infection (unspecified)	-	-	-	1	-	**-**	**-**	**-**	**-**	**-**	** -**
**Other**											
Disease X	-	-	-	-	1	**-**	**-**	**-**	**-**	**-**	** -**

OPG: Olympic and Paralympic Games; WHO: World Health Organization.

^a^ Infectious disease responsible for epidemics at previous OPGs, from the 2002 Winter OPG in Salt Lake City to the 2020 Summer OPG in Tokyo.

^b^ Lassa fever only.

^c^ Dengue, chikungunya and Zika virus disease are classified as ‘zoonoses’, though the viruses are believed to be maintained primarily in human/mosquito cycles, especially in urban areas.

Infectious diseases highlighted in bold correspond to those identified specifically by Santé publique France as being of potential interest for public health surveillance during the Games.

MERS-CoV 2: Middle East respiratory syndrome coronavirus; SARS-CoV-1: severe acute respiratory syndrome coronavirus 1; SARS-CoV-2: severe acute respiratory syndrome coronavirus 2; SpF: Santé publique France.

Of the 62 experts invited to take part in the about 3-month long Delphi exercise, 33 (53%) responded to rounds 1 and 2 and 32 (52%) completed all three rounds of the survey. Among the latter, 26 have a professional background in public health, specialising in infectious diseases, 12 in WBE, 9 in microbiology and 4 in drinking water and/or wastewater treatment. Of note, some experts have expertise in multiple fields.

Five pathogens (or categories of pathogens) meeting the analytical feasibility criterion (A) were also proposed by the panel for evaluation: non-polio Enteroviruses, *Cryptosporidium spp.,* *Giardia duodenalis,* Alphacoronavirus and Betacoronavirus. Consequently, a total of 30 pathogens were assessed against the relevance (B) and added value (C) criteria. At the end of the third Delphi round, group consensus (70%) was reached for 25 pathogens/diseases: five for inclusion and 20 for exclusion from WWS for the Paris 2024 OPG (Table 2). A summary of consensus reached per pathogen per round is available in Supplementary Table S2.

**Table 2:** Summary of results from evaluation of 30 potential pathogens for wastewater surveillance during the Paris 2024 Olympic and Paralympic Games following the 3-round Delphi survey, France, May−July 2023

Potential wastewater surveillance targets		Inclusion criteria			Pathogens retained	Pathogens excluded	Pathogens for which no consensus was found
Pathogen genus/disease	Pathogen species/genogroup	Pertinence		Plus-value to inform public health action			
		Specificities of the event	Pathogen-/disease-specific characteristics				
**Vaccine- preventable diseases**							
Measles	Measles virus (MV)	NA	NA	NA	-	-	1
Poliomyelitis	Poliovirus	Yes	Yes	Yes	1	-	-
**Food- and waterborne diseases**							
Adenovirus	Human adenovirus-F (HAdV-F)	No (39%; 11/28)	No (46%; 13/28)	No (75%; 21/28)	-	1	-
Amoebiasis	*Entamoeba histolytica*	No (56%; 14/25)	No (52%; 13/25)	No (56%; 14/25)	-	1	-
*Campylobacter* infections	17 species have been identified, of which *C. jejuni* and *C. coli* are most frequently reported in human disease	No (37%; 10/27)	No (56%; 15/27)	No (74%; 20/27)	-	1	-
Cholera	*Vibrio cholerae*	NA	NA	NA	-	-	1
*Cryptosporidium*	*C. hominis, C. parvum, C. meleagridis, C. muris*	No (42%; 10/24)	No (46%; 11/24)	No (75%; 18/24)	-	1	-
*Escherichia coli *infections	*Escherichia coli*	No (48%; 14/29)	No (72%; 21/29)	No (76%; 22/29)	-	1	-
*Giardia*	*G. duodenalis*	No (39%; 11/28)	No (50%; 14/28)	No (68%; 19/28)	-	1	-
Hepatitis A	Hepatitis A virus (HAV)	NA	NA	NA	-	-	1
Hepatitis E	Hepatitis E virus (HEV)	No (32%; 9/28)	No (36%; 10/28)	No (82%; 23/28)		1	
Legionnaires’ disease	20 species have been documented as human pathogens, of which *L. pneumophila* most commonly causes of illness humans	No (44%; 11/25)	No (56%; 14/25)	No (64%; 16/25)	-	1	-
Non-polio enterovirus	Enterovirus A, B, C and D	No (44%; 11/25)	No (28%; 7/25)	No (84%; 21/25)	-	1	-
Norovirus	GI, GI1 and GIV	NA	NA	NA	-	-	1
Rotavirus	Rotavirus A	No (33%; 8/24)	No (58%; 14/24)	No (71%; 17/24)	-	1	-
Salmonellosis (Nontyphoidal *Salmonella*)	*Salmonella bongori, Salmonella enterica*	No (41%; 12/29)	No (72%; 21/29)	No (83%; 24/29)	-	1	-
Shigellosis	*Shigella sonnei, Shigella flexneri, Shigella boydii, Shigella dysenteriae*	NA	NA	NA	-	-	1
Yersiniosis	*Yersinia enterocolitica*	No (44%; 12/27)	No (48%; 13/27)	No (59%; 16/27)	-	1	-
**Respiratory diseases**							
Alphacoronavirus	HCoV-229E	No (26%; 7/27)	No (59%; 16/27)	No (74%; 20/27)	-	1	-
Betacoronavirus	Betacoronavirus 1, HCoV-HKU1	No (26%; 7/27)	No (63%; 17/27)	No (67%; 18/27)	-	1	-
Influenza	Influenza A virus	Yes	Yes	Yes	1	-	-
	Influenza B virus	Yes	Yes	Yes	1	-	-
Tuberculosis	*Mycobacterium tuberculosis* complex	No (61%; 14/23)	No (70%; 16/23)	No (48%; 11/23)	-	1	-
**Emerging and vector-borne diseases**							
COVID-19	SARS-CoV-2	Yes	Yes	Yes	1	-	-
Ebola	Zaire ebolavirus (EBOV), Sudan ebolavirus (SUDV), Tai Forest ebolavirus (TAFV), Bundibugyo ebolavirus (BDBV)	No (56%; 14/25)	No (80%; 20/25)	No (80%; 20/25)	-	1	-
Mpox	MPXV	Yes	Yes	Yes	1	-	-
Severe acute respiratory syndrome (SARS)	SARS-CoV-1	No (28%; 7/25)	No (68%; 17/25)	No (40%; 10/25)	-	1	-
**Zoonoses**							
Echinococcosis	*Echinococcus multilocularis*	No (53%; 16/30)	No (73%; 22/30)	No (37%; 11/30)	-	1	-
Leptospirosis	64 species have been identified	No (44% ;11/24)	No (63%; 15/24)	No (58%; 14/24)	-	1	-
**Sexually transmitted infections**							
Human immunodeficiency virus (HIV) infection	Human immunodeficiency virus 1 (HIV-1), Human immunodeficiency virus 2 (HIV-2)	No (50%; 15/30)	No (60%; 18/30)	No (70%; 21/30)	-	1	-
**Total**					**5**	**20**	**5**

COVID-19: coronavirus disease; NA: not applicable.

Where group consensus resulted in the exclusion of pathogens, the cumulative responses of experts regarding the criterion/criteria influencing this decision are enclosed within brackets. Since only the experts who voted for exclusion were asked this question, the denominator reflects the number of experts who voted accordingly. A summary of consensus reached per pathogen per round is available in Supplementary Table S2.

Additionally, five pathogens (measles virus, hepatitis A virus, *Vibrio cholerae*, norovirus and *Shigella* spp.) failed to reach the consensus threshold (70%). Given the increased risk of importation, transmission and dissemination of pathogens during the Games [6] and the notable increase in measles cases worldwide in 2023 and in Europe at the time of evaluation (February 2024) [19,20], measles virus was retained as a WWS target for Paris 2024. Moreover, the virus was only 1% (22/32) away from group consensus for inclusion in the final Delphi round. Conversely, hepatitis A virus and *Vibrio cholerae* were 1% (22/32) and 4% (21/32) away from exclusion, respectively. Group positions on norovirus and *Shigella* spp. were more evenly distributed (Table 3).

**Table 3:** Expert opinion on five pathogens failing to reach the 70% consensus threshold for wastewater surveillance during the Paris 2024 Olympic and Paralympic Games following the third round of the Delphi survey, France, May−July 2023 (n = 32 experts)

Pathogen	Inclusion		Exclusion	
	%	n	%	n
Measles virus	69	22	31	10
*Vibrio cholerae*	34	11	66	21
Norovirus	56	18	44	14
*Shigella* spp.	44	14	56	18
Hepatitis A virus	31	10	69	22

Ultimately, the other four were excluded based on their failure to satisfy the relevance and added value criteria. *Vibrio cholerae* WWS seems of little relevance in a high-income country such as France, characterised by access to safe chlorinated drinking-water, good sanitary conditions, and free healthcare. Indeed, in this setting, secondary transmission of *Vibrio cholera* is unlikely [21,22]. Noroviruses and *Shigella* spp. frequently circulate both in France and abroad [23,24]. Thus, detection would neither identify the source nor trigger specific public health measures. Finally, the lengthy incubation period for hepatitis A (15–50 days) limits the relevance of its wastewater monitoring within the brief timeframe of the Games [25]. A flowchart summarising the steps taken to identify priority pathogen targets for the 2024 OPG is available as Figure 2. The most relevant WWS objectives for pathogens retained are presented in Table 4.

**Table 4:** Expert opinion on the most relevant surveillance objectives for the six priority pathogen targets identified for wastewater surveillance during the Paris 2024 Olympic and Paralympic Games, France

Pathogen	Detection (presence/absence)	Quantification	Trend monitoring	Assessment of genetic diversity
Poliovirus	100%	29%	21%	68%
Influenza A virus	52%	65%	82%	65%
Influenza B virus	56%	61%	65%	56%
Mpox virus	86%	53%	71%	29%
SARS-CoV-2	47%	70%	90%	87%
Measles virus	95%	23%	50%	32%

SARS-CoV-2: severe acute respiratory syndrome coronavirus 2.

The data presented correspond to the responses of the sub-section of participants who voted for these pathogens to be included as targets for WWS during the Paris 2024 Olympic and Paralympic Games either: (i) in the Delphi round in which consensus was reached for their inclusion (for pathogens retained) (Supplementary Table S2) or (ii) at the end of the third round of the Delphi survey (for pathogens which failed to reach group consensus, but were retained, i.e. measles virus).

Four diseases whose causative agents did not satisfy the analytical feasibility criterion were also proposed by the panel for assessment: tetanus (*Clostridium tetani*), plague (*Yersinia pestis*), tick-borne encephalitis (tick-borne encephalitis virus) and listeriosis *(Listeria monocytogenes*). These were added to the list of prospective pathogens for wastewater research. Those most frequently selected as research priorities across Delphi rounds were dengue virus (DENV), chikungunya virus (CHIKV) and West Nile virus (WNV), each receiving an average of 14, 14 and 11 votes, respectively. A full overview of expert opinion for this question is available in Supplementary Figure S1.

## Discussion

To our knowledge, this is the first study that uses expert opinion to prioritise pathogens for WWS during an international MG such as the OPG. The framework used identified six priority targets suitable for WWS during the Paris 2024 OPG: poliovirus, influenza A virus, influenza B virus, mpox virus, SARS-CoV-2 and measles virus. Expert opinion on pathogen-specific surveillance objectives such as detection, quantification, trend monitoring and assessment of genetic diversity was also gathered throughout the Delphi exercise. Such information is needed to frame the wastewater sampling strategy for the Games, notably with regards to sampling locations and frequencies.

The pertinence of priority targets identified highlights the strength of the method. Wastewater surveillance is one of the main tools recommended by the Global Polio Eradication Initiative (GPEI) to detect and monitor poliovirus circulation, including in polio-free countries such as France. Detection of the virus in such countries can act as an early warning system [3]. In 2022, the detection of type 2 vaccine-derived poliovirus in sewage samples in London, United Kingdom and New York City, United States (US) underscored the value of this surveillance [5,26]. It enabled the identification of the geographical area of virus circulation and informed the implementation of public health measures, e.g. enhanced surveillance, vaccination campaigns, etc.

Influenza A and B viruses represent a relevant public health burden, with worldwide distribution and year-round circulation. Epidemics typically occur between November and April in the northern hemisphere, and between June and October in the southern hemisphere [27]. Wastewater surveillance has been shown to accurately reflect the seasonal onset and prevalence of influenza, even outperforming clinical surveillance [28]. Integrating WWS during the 2024 OPG could provide valuable insight into the background circulation of influenza A and B viruses in Paris during summer months and facilitate tracking of potential transmission chains of the viruses imported by travellers from the southern hemisphere. Elevated virus titres in wastewater could, in turn, prompt increased testing and diagnosis of influenza cases, which might otherwise be misdiagnosed. For instance, an influenza case could be mistaken for a COVID-19 case if SARS-CoV-2 circulation is ongoing.

Wastewater surveillance has been found to have relatively high sensitivity to detect even a small number of cases of mpox virus infections [29] and the potential to provide early detection of virus circulation in non-endemic countries [30]. Further, wastewater data can be used alongside clinical surveillance data to deliver better estimates of the scale of epidemics. Mpox virus has been detected in wastewater both at disproportionately high levels when compared with identified cases, as well as in the absence of reported cases [31]. Indeed, the social stigma related to the higher incidence of mpox among men who have sex with men (MSM) is believed to contribute to its underdiagnosis [30,31]. This trend may be exacerbated among foreign tourists during the OPG.

Wastewater surveillance of SARS-CoV-2 has proven useful in informing public health actions during the COVID-19 pandemic, notably by: (i) validating trends observed through other data sources; (ii) informing the allocation of resources, e.g. personnel, hospital beds, screening sites, vaccination sites; and (iii) guiding the implementation of prevention and control measures, e.g. mask wearing, social distancing [1,4]. Finally, measles virus was deemed of special interest for WWS during the Games given the epidemiological situation in Europe and internationally at the time of evaluation (February 2024) [19,20] and increased risk of virus circulation during the Games [6].

Wastewater-based epidemiology is a field that has developed considerably since 2020, though many uncertainties remain. While it has been shown that multiple variables including pathogen-specific structural characteristics (presence of a lipid envelope, affinity with organic matter), and environmental parameters (temperature, pH and biological activity) affect the persistence of pathogens in the environment, our understanding of pathogen shedding dynamics and stability in wastewater remains limited [15]. To account for this, we considered the analytical feasibility criterion met if there was published evidence that a given pathogen has been detected in wastewater previously. Nonetheless, it is possible that pathogens of interest were excluded based on the wrongful assumption that they cannot be detected in wastewater. Potential pathogens should be reviewed in the light of the global health situation and new emerging diseases should be considered. The example of mpox is very illustrative in this respect, with a recent (re)emergence in 2022–23 followed by a prompt adaptation of WWS systems, notably in the United States [32]. We recommend that this criterion be reassessed regularly as knowledge in the field progresses.

The solicitation of expert opinion via the Delphi method is useful in areas where knowledge is incomplete and objective data are unavailable [17,18]. Although judgements made based on the collective opinions of the members of a group are often better than those made by a single member, relying on expert opinion can present certain limitations. Firstly, the quality of conclusions is limited by the expertise of panel members. Further, the success of the Delphi process depends on the impartial judgment of participants [17]. For this reason, particular attention was paid to the choice of experts invited to take part in this exercise, especially with regard to their potential conflicts of interest and qualifications. Although WBE is an interdisciplinary field, determining whether a given pathogen is a suitable WWS target for the OPG is fundamentally a public health question. This explains the high proportion of public health experts who made up our expert panel (26/32). Further, special care was taken to ensure that panellists selected for their experience in microbiology and drinking-water/wastewater treatment also possessed competencies in public health or WBE, allowing them to fully leverage their knowledge within the project’s scope. This, along with the initial pathogen screening based on analytical feasibility carried out prior to the Delphi exercise, justifies the smaller representation of microbiology (9/32) and drinking-water/wastewater treatment (4/32) professionals among panellists.

Studies have shown that Delphi groups that receive feedback on why participants opted for certain responses in addition to quantitative analyses of respondent data reach more accurate conclusions than groups that receive only response statistics [18]. Consequently, experts were given the opportunity to leave comments at every step of the survey process. This information was summarised and provided to all participants at the start of each new round.

This pathogen prioritisation exercise and the information gathered throughout its course aimed to serve as a starting point for a WWS plan for the Paris 2024 OPG. However, more work is needed before an implementation. In France, WWS operates through the SUM'Eau network, which monitors SARS-CoV-2 circulation in 54 wastewater treatment plants nationwide. A WWS plan for the Games would leverage this existing system. At the time of writing, a wastewater sampling strategy proposal based on the conclusion of this study was under review by the French health authorities. Upon approval, the next steps will include the selection of operators in charge of sampling and partner laboratories that will perform analyses. For certain pathogens, laboratory methods will need to be developed for detection and quantification. Methods will also need to be optimised in terms of time efficiency and cost-effectiveness. In addition, technical feasibility constraints may necessitate adjustments to the sampling strategy and could warrant further refinement of pathogen targets and/or WWS objectives. A WWS plan for Paris 2024 OPG would serve as an exploratory tool, providing proof of concept for monitoring measles virus, influenza A virus and influenza B virus in Parisian wastewaters.

In considering possible future research directions, the Delphi rounds also collected expert opinion on which of the 29 pathogens excluded based on a lack of evidence of detectability in wastewater (analytical feasibility criterion), should constitute research priorities. Among the most frequently selected (DENV, CHIKV and WNV), all were arboviruses. This can be explained by the increasing risk of mosquito-borne diseases in Europe following the spread of *Aedes* species. The impact of climate change on the continent has been marked with more frequent heat waves and flooding as well as longer and warmer summers. These changing weather patterns are creating favourable conditions for invasive mosquito species like *Aedes albopictus* (known to transmit DENV, CHIKV, yellow fever virus and Zika virus) to thrive [33].

Over the last few decades, the World has seen a global rise in human infectious disease outbreaks [34]. Environmental changes themselves linked to anthropogenic activities are modifying ecosystems’ structure and composition as well as interactions between host, pathogen, and the environment. Human behaviour, demographic changes, globalisation, and international travel are bringing people in closer and more frequent contact with pathogens, facilitating their dissemination. In addition, the evolution and selection for resistant microorganisms suggest that communicable diseases will continue to spread and emerge [35,36]. Given the inevitability that pathogens with epidemic potential will threaten public health again, the development of new cost-effective public health monitoring tools, such as WWS, seems not only relevant, but essential.

## Conclusion

This study introduced a model framework for identifying context specific WWS targets for an MG event. A successful WWS plan for the Paris 2024 OPG and the lessons learned from its design and implementation should have a notable impact on the development of the field of WBE. Notably, it could facilitate the extension of France’s routine WWS to other pathogens, and act as both an incentive and a starting point for the implementation of such surveillance during other MGs globally.

## Supplementary Data

787000 Supplement
