# Supplementary material for: Pathogen prioritisation for wastewater surveillance ahead of the Paris 2024 Olympic and Paralympic Games, France
Source: Euro Surveill. 2024 Jul 11;29(28):2400231. doi: 10.2807/1560-7917.ES.2024.29.28.2400231 (PMC11241851; doi:10.2807/1560-7917.ES.2024.29.28.2400231)

This supplementary material is hosted by *Eurosurveillance* as supporting information alongside the article "**Pathogen prioritisation for wastewater surveillance ahead of the Paris 2024 Olympic and Paralympic Games, France**", on behalf of the authors, who remain responsible for the accuracy and appropriateness of the content. The same standards for ethics, copyright, attributions, and permissions as for the article apply. Supplements are not edited by *Eurosurveillance* and the journal is not responsible for the maintenance of any links or email addresses provided therein

**Supplement S1:** *Example of the search query used to evaluate analytical feasibility adapted to Vibrio cholerae*

(Cholera[TIAB] OR Vibrio cholerae[TIAB] OR V. cholerae[TIAB])) AND (wastewater*[TIAB] OR waste water*[TIAB] OR sewage*[TIAB] OR sludge[TIAB] OR sewer water[TIAB]) AND (detection*[TIAB] OR detect*[TIAB] OR monitoring[TIAB] OR surveillance[TIAB])

**Supplement S2:** Example of questions asked for the 30 pathogens evaluated throughout the Delphi survey, adapted to Measles virus

For each pathogen evaluated, a participant was asked either [Q2] or [Q3], depending on his/her answer to [Q1]

**Measles virus**

*[Q1] In your opinion, is wastewater surveillance during the Paris 2024 Olympic and Paralympic Games (OPG) a suitable method for monitoring the following pathogen:* ***Measles virus***

***Note:*** *To be a good wastewater surveillance target in this context, the surveillance of this pathogen must:*
(1) be **relevant** with regard to the specificities of the **event**
(2) be **relevant** with regard to the specificities of the **pathogen** and the associated **disease**(s), and
(3) **add value** to inform public health decision making with regard to population-based surveillance already in place (i.e. nationally notifiable diseases, sentinel surveillance, emergency department admission rates, screening tests data, etc.)

Please select one of the following options:

- ☐ Yes
- ☐ No

---

***Question [Q2] was only asked if the following condition was met:** The answer to question '[Q1]' was 'Yes'.
[Q2] *Which of the following would be the most appropriate wastewater surveillance objective(s) for* ***measles virus*** *during the Paris 2024 OPG?*

Please check all that apply:
- ☐ Detection (presence/absence);
- ☐ Quantification
- ☐ Trend monitoring
- ☐Assessment of genetic diversity

**Comment(s)**

| |
|---|
| |

---

***Question [Q3] was only asked if the following condition was met:** The answer to question '[Q1]' was 'No'.
*[Q3] Based on which criterion(s) do you think* ***measles virus would not be a good target*** *for wastewater surveillance during the Paris 2024 OPG?*

Please check all that apply:
- ☐ **Irrelevance** with regard to the specificities of the **event**
- ☐ **Irrelevance** with regards to **pathogen-** /**disease-** specific characteristics
- ☐ Lack of **added value** to inform public health decision-making

**Comment(s)**

| |
|---|
| |

**Supplementary Table S1**: Key information on potential pathogens for WS during the Paris 2024 Olympic and Paralympic Games

| # | Potential pathogen / disease targets for wastewater surveillance | Pathogen species / genogroup | Pathogen subcategory (Serotype / Strain / Sub-species / Genotype / Assemblage) | Key information on potential pathogens: Detectability in wastewater | Incubation period | Period of communicability | Duration pathogen shedding in urine/feces | Mode of transmission | Seasonality of outbreaks | Occurrence | Existing surveillance in France | Amount of pathogen circulation expected in metropolitan France (independantly of the games) | Host range | Sources detectability in wastewater | Sources other |
|---|---|---|---|---|---|---|---|---|---|---|---|---|---|---|---|
| | **Vaccine- preventable diseases** | | | | | | | | | | | | | | |
| 1 | Meningococcal disease | *Neisseria meningitidis* | | No evidence | | | | | | | | | | | |
| 2 | Measles | Measles virus | | Yes | 8 to 12 days | 4 days prior to the rash onset to 4 days after the rash erupts - rash onset usually occurs 14 days after initial infection | Has been detected in urine up to 10 days after the onset of rash in immunocompetent patients | Airborn by the spread by respiratory droplets and by direct contact with nasal or throat secretions from an infected person. Less commonly transmission can occur via indirect contact with contaminated objects. | | Worldwide distribution, although measles cases occur primarily in developing countries, particularly in Africa and Asia. | 2 | If the trends continue, circulation is possible. Historically, measles cases are rare in Europe and the Americas, with outbreaks being sporadic due to imported cases and unvaccinated populations. However, the European Union saw a significant increase in cases in 2023. Measles vaccination is mandatory in France. | Humans (primary host), non-human primates | *[1]* | *[2–7]* |
| 3 | Diphtheria | *Corynebacterium diphtheriae* | | No evidence | | | | | | | | | | | |
| 4 | Whooping cough | *Bordetella pertussis*<br>*Bordetella parapertussis* | | No evidence | | | | | | | | | | | |
| 5 | Poliomyelitis | *Poliovirus* | Type 1 wild poliovirus (WPV1)<br>Circulating vaccine-derived polioviruses types 1 to 3 (cVDPV1, cVDPV2 and cVDPV3) | Yes | Usually 7 to 10 days, but can range from 4 to 35 days | Exact period has not been defined, but humans can transmit the infection as long as virus is excreted. Virus has been detected in throat secretions and feces 36 hours and 72 hours after infection, respectively. | Virus persists in the throat approximately 1 week and in feces for 3 to 6 weeks, sometimes longer | Person-to-person primarily through the fecal-oral route, in particular via contaminated water, aerosols or food | In temperate climates, cases occur primarily during summer and autumn. In tropical areas, the seasonal pattern is less pronounced. | Wild poliovirus type 2 and wild poliovirus type 3 were eradicated in 1999 and 2020, respectively. As of 2022, wild poliovirus type 1 remains endemic Pakistan and Afghanistan. Circulating vaccine-derived polioviruses (cVDPV types 1, 2 and 3), have an nearly worldwide distribution with cases idenfied in over 50 countries from 2020 to feburary 2023. | 2, 5 | None. Vaccination is mandatory in France since january 1st 2018. No cases of vaccine-associated paralysis have been reported since 1983, and no wild indigenous cases of paralytic poliomyelitis have been reported since 1990. However, Sabin-like type 2 poliovirus (SL2) was detected in environmental samples in the United Kingdom and the United States of America in 2022. | Humans | *[8,9]* | *[2,10–18]* |
| 6 | Rubella | *Rubivirus rubellae (Rubella virus)* | | No evidence | | | | | | | | | | | |
| 7 | Typhoid fever (Typhoidal Salmonella) | *Salmonella enterica* | Typhi | No evidence | | | | | | | | | | | |
| 8 | Tetanus | *Clostridium tetani* | | No evidence | | | | | | | | | | | |
| | **Food- and waterborne diseases** | | | | | | | | | | | | | | |
| 9 | *Escherichia coli* infections | *Escherichia coli* | There are 6 pathotypes of diarrheagenic *E. coli*: Shiga toxin-producing *E. coli* (STEC), Enterotoxigenic *E. coli* (ETEC), Enteropathogenic *E. coli* (EPEC), Enteroaggregative *E. coli* (EAEC), Enteroinvasive *E. coli* (EIEC) and Diffusely adherent *E. coli* (DAEC) | Yes | From a few hours to 8 days, depending on strain.<br><br>STEC : 3 to 8 days, usually 3 to 4 days | Infected persons can spread the disease for as long bacteria is shed in their feces | STEC fecal shedding usually persists for 1 week amongst adults but is longer for children | STEC transmission is primarily indirect through the consumption of contaminated food (ex: raw or undercooked ground meat products and raw milk) or water. Direct person-to-person contact through the fecal-oral route can also cause infections. | | Worldwide distribution. In 2021, 6 534 confirmed cases of STEC infections were reported amongst the 30 EU/EEA countries, 298 of which occurred in France. | 2*, 3, 8* | Some circulation is likely. | Humans, piglets, calves and cattle | *[19]* | *[20–23]* |
| 10 | Cholera | *Vibrio cholerae* | O1<br>O139 | Yes | From a few hours to 5 days, usually 2 to 3 days | Infected persons can spread the disease for as long bacteria is shed in their feces | Pathogen shedding usually persists for 1 to 10 days after infection, but in some cases can last several months | Cholera is spread primarily by ingestion of an infective dose through contaminated water (fecal-oral route). However, epidemics caused by contaminated foods such as raw fish and seafood have been reported. | Seasonal peaks of the disease occurr during the warmer months, when conditions (water temperature and salinity) are optimal for the bactrial growth. | Cholera is endemic in approximately 50 countries, primarily in Africa and South and Southeast Asia. | 2, 3 | None. Chloera is a rare disease in France. Between 2010 and 2018, 16 cases were reported in the country, most of which were imported from North America (Haiti) and Asia. | Humans, water birds, shellfish, fish, and herbivores | *[24–27]* | *[2,28–32]* |
| 11 | Salmonellosis (Nontyphoidal | *Salmonella bongori* | > 2,500 Salmonella serotypes have been | Yes | 5 to 72 hours, depending on the inoculum size | Infected persons can spread the disease for | Pathogen shedding can last from several days to several months. In 1% of infected | Transmission occurs through the consumption of food or water contaminated with the feces of infected humans or animals (fecal-oral route). The | Human salmonella infections generally | Worldwide distribution. Nontyphoidal salmonellae are | 2*, 3, 8* | Some circulation is likely. In France, the CNR, isolates roughly 8,000 to 11,000 cases of Salmonella each year, with 8,793 cases in 2020. Indeed, the | Depends on the serotypes, although most are present in a wide range of hosts, including : humans ; domestic and wild | *[26,33]* | *[2,34–40]* |

| | | | | | | | | | | | | | | | |
|---|---|---|---|---|---|---|---|---|---|---|---|---|---|---|---|
| [illegible] | [illegible] Salmonella) | *Salmonella enterica* | [illegible] identified | [illegible] | [illegible] Usually 6 to 72 hours | [illegible] as long bacteria is shed in their feces | [illegible] adulting and 5% of infected children, shedding can persist for over 1 year. | [illegible] disease can also spread through direct contact with infected animals or their environment and directly from person-to-person. | [illegible] peak in summer months | [illegible] one of the four leading causes of bacterial diarrhea. | [illegible] | [illegible] incidence of human salmonellosis in the country is estimated at approximately 307 cases per 100,000 inhabitants per year. | [illegible] animals such as cattle, swine, poultry, wild birds ; and in pets, including cats, dogs, birds, and reptiles such as turtles | [illegible] | [illegible] |
| **12** | *Norovirus* | *GI*<br>*GII*<br>*GIV* | | Yes | 15 to 48 hours | From the onset of symptoms until at least 3 days after recovery, sometimes persisting for up to two weeks | Viral shedding usually persists in the feces and vomitus of symptomatic and asymtomatic patients for 10 to 28 days. In immunocompromised patients however, virus can be shed in feces for months after an infection. | Transmission is primarily person-to-person through the fecal-oral route, but can also occur through the environment via contaminated surfaces, food, water, fomites, and aerosols. | Outbreaks peak in winter months but can occur throughout the year | Worldwide distribution. | 2*, 3, 8* | Some circulation is likely. Noroviruses are responsible for a third of all foodborne infections in France, with an estimated 516,000 cases per year. | Humans, pigs, cattle, mice | *[41–46]* | *[47–52]* |
| **13** | *Rotavirus* | *Rotavirus A* | | Yes | 1 to 2 days | Begins 2 days before the onset of diarrhea and persists for several days | Viral shedding in stool begins 2 days before the onset of diarrhea and persists for several days. In immunocompromised patients however, virus can be excreted for more than 30 days after an infection. | Transmission is primarily person-to-person through the fecal-oral route, but can also occur through contaminated objects (fomites), airborne droplets and contaminated water or food. | In europe, rotaviruses outbreaks cause peak between December and May, however, sustained low-grade transmission is observed all year round. | Worldwide distribution. The virus is one of the main causes of severe diarrhea in children. Rotavoirus infections cause roughly 125,000–200,000 deaths every year, half of which occur in just four countries : India, Nigeria, Pakistan, and the Democratic Republic of the Congo. | 2*, 3, 8* | Some circulation is possible. Every year in France, noroviruses are responsible for roughly 20,000 hospitalizations in children under the age of 3 and 60,000 medical consultations. | Humans, cows, goats, wild animals, ostriches, chicken, dogs, and horses | *[53–55]* | *[56–63]* |
| **14** | *Adenovirus* | *Human adenovirus-F (HAdV-F)* | Serotype 40<br>Serotype 41 | Yes | 3 to 10 days | Infected persons can transmit the pathogen for as long as viral shedding persists | Virus shedding in stool takes place during the acute stage of the disease and usually last 7 to14 days, but can persist for longer among people with weakened immune systems. Asymptomatic individuals may also release adenovirus in their stool. | Transmission is person-to-person via the fecal-oral route often through consumption of contaminated drinking water | No clear seasonality has been identified. | Worldwide distribution. They are the third most common cause of gastroenteritis in children after rotavirus and norovirus. | 3, 8* | Some circulation is likely. | Humans | *[45,46,53,64,65]* | *[2,66–72]* |
| **15** | Campylobacter infections | 17 species have been identified, of which *C. jejuni* and *C. coli* are most frequently reported in human disease | | Yes | 1 to 10 days, usually 2 to 5 days | Person-to-person transmission is possible but rare. Infected persons can transmit the bacteria throughout the course of infection, usually several days to weeks. | Bacterial shedding lasts 2 to 3 weeks, but can persist for up to 7 weeks. | Transmission is primarily indirect through the consumption of contaminated food (ex: undercooked meat (primarily chicken) and unpasteurized milk) or water. Direct animal-to-person or person-to-person transmission has also been described, but is less frequent. | In temperate areas a sharp increase in cases are observed in summer months. | Worldwide distribution. Campylobacter is the most common bacterial causes of human gastroenteritis. The pathogen is also 1 of 4 key causes of diarrhoeal disease, causing 5-14% of all diarrhea cases worldwide. | 2*, 3, 8* | Some circulation is likely. In France, Campylobacter represents 26% of all foodborne infections, with an incidence of 25 cases per 100,000 inhabitants in 2020. | Large host range including: pig, poultry, cattle, sheep, rabbits, mink, dogs, cats, avian animals and humans | *[73]* | *[74–80]* |
| **16** | Shigellosis | *Shigella sonnei*<br>*Shigella flexneri*<br>*Shigella boydii*<br>*Shigella dysenteriae* | | Yes | Usually 1 to 3 days, but can range from 12 hours to 7 days | Infected persons can transmit the pathogen during acute infection and for as long as the pathogen is released in their stool | Fecal shedding usually persists for 4 weeks after illness. | Transmission occurs through the fecal-oral route via ingestion of contaminated food or water, or person-to-person contact (ex: through anal sexual contact). Flies can also acts as mechanical vectors for disease spread. | In temperate regions, incidence peaks during summer and early fall. In tropical regions cases spike during the rainy season. | Worldwide distribution. 5 to 15% of all diarrhea cases are due to shigella spp. infection. | 3 | Some circulation is possible. In 2019, the CNR detected 8 *S. sonnei* and one *S. flexneri* outbreaks in France and identified over 1,300 strains of the bacteria. | Humans, monkeys and apes | *[26]* | *[2,81–84]* |
| **17** | Amoebiasis | *Entamoeba histolytica* | | Yes | From several days to several months, usually 2 to 4 weeks | Infected persons can transmit the pathogen for as long as E. histolytica cysts are shed in feces | Fecal shedding can persist for years after infection. | Transmission can occur through fecal-oral route via the ingestion of contaminated food and water containing amebic cysts. It may also occur sexually by oral-anal contact with an infected person. | Different seasonal trends have been reported in different regions. | Worldwide distribution, however, E. histolytica is more common in tropical and subtropical areas (ex: Mexico, South and West Africa, western South America and South Asia) and in temperate areas with poor sanitation. The pathogen is endemic in Mexico, the Indian subcontinent, Indonesia, sub-Saharan and tropical regions of Africa, and parts of Central and South America. Although 90% of infections are asymptomatic, Entamoeba histolytica is the second leading cause of parasitic death in the world. | 1 | Some circulation is possible. In metropolitan France there is no data on the pathogen in the general population. | Humans and non-human primates | *[85,86]* | *[2,87–92]* |

| | | | | | | | | | | | | | | | |
|---|---|---|---|---|---|---|---|---|---|---|---|---|---|---|---|
| **18** | Hepatitis A | *Hepatitis A virus (HAV)* | | Yes | 15 to 50 days (28 to 30 days on average) | Infected persons can transmit the pathogen for as long as viral shedding persists. Peak infectivity occurs during the latter half of the incubation period and continues for a few days after the onset of symptoms. | Levels of virus in feces peak 1 to 2 weeks prior to the onset of symptoms, and diminish quickly after symptoms appear. Prolonged viral shedding has been observed for up to 6 months. | Transmission is person-to-person through the fecal-oral route. Contanimated food and water are most common source of infection. Other potential sources of infection include sexual contact, blood transnfusions, and both injection and non-injection drug use. | Consistent seasonality has not been proven, but the literature points towards peaks in cases in spring and summer months | Worldwide distribution, though the highest seroprevalence rates are observed in Central and South America, Africa, India, the Middle East, and Asia and the lowest are seen in North America, Japan, and Western Europe. | 2, 3 | Some circulation is possible. Roughly 1 thousand cases of hepatitis A are reported in France every year, mostly among people returning from countries where virus circulation is high. | Humans, chimpanzees and other non-human primates | *[41,53,93,94]* | *[2,95–99]* |
| **19** | Hepatitis E | *Hepatitis E virus (HEV)* | | Yes | 15 to 60 days (40 days on average) | The specific period of infectiousness for HEV is unknown | Viral shedding in stool has been observed from 1 week prior to 30 days after the onset of jaundice. Chronically infected persons release HEV as long as they remain infected. | HEV can be spred through : fecal-oral transmission, food-borne transmission, blood-borne transmission, and vertical transmission. | Consistent seasonality has not been proven, but the literature points towards peaks of cases in spring and summer months | Worldwide distribution but is most common in East and South Asia. | 2*, 3 | Some circulation is possible. HEV is endemic in France, with an average prevalence of around 20%, and hyperendemic in some areas. For instance, in Southern France over 86% of the blood donors are found to be HEV positive. | Humans, non-human primates, cows, sheep and goats | *[94,100,101]* | *[2,102–104]* |
| **20** | Legionnaires' disease | *20 species have been documented as human pathogens, of which L. pneumophila most commonly causes of illness humans* | | Yes | 2 to 10 days, usually 5 to 6 days | No person-to-person, animal-to-perspon, nor animal-to-animal transmission has been documented | Pathogen shedding has been demonstated in both urine and feces. One study found Legionella antigen could be detected as early as 1 day after onset of symptoms and persists for days to weeks. Antigen was detected 42 days or longer after the begining of treatment in at least 15 patients. In one instance, excretion persisted for 326 days. | Airborn transmission by aerosols or by aspiration of contaminated water. | Incidences increase in the summer and fall months, though infections occur year round. | Worldwide distribution, with cases reported in North and South America, Asia, Australia, New Zealand, Europe and Africa. | 2, 6 | Some circulation is possible. In 2019, 1,816 cases of Legionellosis/Legionnaires' disease where reported in France, causing 160 deaths. | Humans, amoeba | *[105–107]* | *[2,108–114]* |
| **21** | Yersiniosis | *Y. enterocolitica* | | Yes | 3 to 10 days | Person-to-person transmission can occur but is rare. Infected persons can transmit the pathogen for as bacterial shedding persists. | Bacteria is shed in feces at least as long as symptoms persist, usually 2 to 3 weeks. If untreated, pathogen shedding can last for as long as 2-3 months. Prolonged asymptomatic carriers have also been reported. | Transmission typically occurs through the consumption of food (ex: raw pork products, undercooked pork, tofu and unpasteurized milk ) or water contaminated with the feces of infected humans or animals (fecal-oral route). Nosocomial transmission and infections related to transfusions of contaminated blood have also been recorded. | Cases peak in fall and winter months but can occur throughout the year | Worldwide distribution, though cases are less common in tropical areas. The highest isolation rates of the pathogen have been reported in temperate and cold regions including: northern Europe, North America and temperate regions of South America. | 2* | Some circulation is possible. *Y. enterocolitica* and *Y. pseudotuberculosis* are the 3rd most common cause of bacterial diarrhea in France and in Europe (after Salmonella and Campylobacter). In France, roughly 30,000 cases occur each year. Indeed, incidence is estimated to be between 2 and 16 cases per 100,000 inhabitants, though this number is believed to be underestimated. | Large host range including: humans, farm animals (ex: sheep, cattle, goats, poultry and pigs), boars, birds and rarely in reptiles, fish and shellfish | *[115]* | *[2,116–121]* |
| **22** | | *Y. pseudotuberculosis* | | No evidence | | | | | | | | | | | |
| **23** | Brucellosis | *B. abortus* | | No evidence | | | | | | | | | | | |
| | | *B. canis* | | No evidence | | | | | | | | | | | |
| | | *B. melitensis* | | No evidence | | | | | | | | | | | |
| | | *B. suis* | | No evidence | | | | | | | | | | | |
| **24** | Botulism | *Clostridium botulinum* | | No evidence | | | | | | | | | | | |
| **25** | Listeriosis | *Listeria monocytogenes* | | No evidence | | | | | | | | | | | |
| | | *Enterovirus A, Enterovirus B, Enterovirus C* | Coxsackie A virus 1–22, 24<br>Coxsackie B virus 1–6 | Yes | Highly variable and will depend on the clinical course of the disease: can last days (ex: hand-foot-and-mouth disease) to years (ex: myocarditis). | Infected persons can transmit the pathogen during the acute stage of illness, maybe longer | Viral shedding in stool can persist for several weeks. | Person-to-person transmission is common and can occur through direct contact with the oral, ocular, respiratory discharges or feces (fecal-oral route) of an infected person, as well as by aerosol droplet spread. | Outbreaks peak in summer and fall months, with sporadic cases year round. | Worldwide distribution. | 3, 5 | Some circulation is possible. Outbreaks have been reported in France and other european countries in the past couple of years. In 2022, during weeks 1 to 22, 79 cases of infection with positive detection of enterovirus in cerebrospinal fluid were reported by the enterovirus surveillance network in France. Coxsackie viruses CV-B4 and CV-B5 were the most commonly detected. | Humans, monkeys, mice | *[122–126]* | *[2,127–130]* |
| | | *Enterovirus B* | Echoviruses 1–7, 9, 11–21, 24–27, 29–33 | Yes | Usually 2 to 10 days | Infected persons can transmit the pathogen for as long as viral shedding persists | Virus is shed in stool for 5 to 6 weeks | Is primarily fecal-oral via respiratory, transplacental, perinatal, and self-inoculation routes. However, the virus may also be transmitted though contact with fomites. | In temperate climates, incidence peaks during the summer and fall months, while in the tropics, cases are reported year-round. | Worldwide distribution. | 3, 5 | Some circulation is possible. Enterovirus infections are identified every year in France, with Echoviruses being amongst those most commonly reported. | Humans | *[122–126]* | *[129–132]* |

| | | | | | | | | | | | | | | | |
|---|---|---|---|---|---|---|---|---|---|---|---|---|---|---|---|
| 1 | Non-polio enteroviruses | *Enterovirus D* | Enterovirus D68 (EV-D68) | Yes | 1 to 5 days | Is believed to be similar to that of rhinoviruses and other enteroviruses and last from 1 day prior to 5 days after the onset of symptoms. | | Transmission is usually fecal-oral or oral-oral and occurs through person-to-person contact or the ingestion of contaminated foods or water. The disease can also spread through aerosols and droplets via coughing, sneezing, talking and breathing. | In temperate regions, enteroviruses circulate more commonly in summer and fall months. Although the seasonality of EV-D68 is not fully understood, outbreaks appear to occur within or later than the typical enterovirus season. Spikes of cases have been observed in late summer and early fall in multiple countries, including the United States, Canada and France. | Worldwide distribution, with cases reported across North America, South America, Europe, Asia, Africa and Australia | 5 | Some circulation is possible. Outbreaks have been reported in several european countries in recent years, and cases seem to be on the rise. A longitudinal study conducted at Lyon University Hospital between 2010 and 2016 showed a two-yearly increase in the circulation of EV-D68. For EV-A71, three epidemics occurred in France in 2013, 2016 and in 2019. | Humans | *[122,126,133–135]* | *[130,136–140]* |
| | | *Enterovirus A* | Enterovirus A71 (EV-A71) | | 3 to 6 days | | Virus is shed in stool for up to 11 weeks after symptoms have subsided | | EV-A71 circulation shows a marked seasonal pattern in the northern hemisphere, with an increase in cases in spring to fall months. This pattern is not observed in tropical areas were the seasonal distribution is homogenous. | | 5 | | | | |
| 2 | *Cryptosporidium* | *C. hominis* | | Yes | 2 to 10 days (7 days on average) | Infected persons can transmit the pathogen for as long as oocytes are shed in feces | Oocysts (the infectious stage) are shed in feces from the onset of symptoms and can persist for up to 50 days after they resolve. | Is fecal–oral through the ingestion of viable oocysts of animal (animal-to-person) or human (person-to-person) origin. Transmission occurs via direct contact or through contaminated food, water and aerosols | In Europe, increased incidence has been described in late spring and in late summer–early autumn. | Worldwide distribution. Cryptosporidium has been identified in over 50 countries and is ranked as the 5th most important outbreak-prone foodborne parasite. Indeed, the pathogen is one of the leading causes of infectious diarrhea globally. From 2011 to 2016, it was the 2nd most common cause of diarrheal disease and death among children in developing countries. | 2*, 3, 8* | Some circulation is possible. In France, data collected by the Expert Laboratory of the Cryptosporidiosis National Reference Center (CNR-LE-Cryptosporidiosis) suggests outbreaks are frequently underreported but common. From 2017 to 2020 11 outbreaks of the disease were identified in the country. | Humans and over 150 species of mammals, fish, amphibians, reptiles, and birds | *[141,142]* | *[2,143–146]* |
| | | *C. parvum* | | | | | | | | | | | | | |
| | | *C. meleagridis* | | | | | | | | | | | | | |
| | | *C. muris* | | | | | | | | | | | | | |
| 3 | *Giardia* | *G. duodenalis* | Assemblage A | Yes | 3 to 25 days (7 to 10 days on average) | The perios od communicability is highly variable but often lasts for several months. Infected persons can transmit the pathogen for as long as cysts are shed in their feces. The pathogen is most contagious during the diarrheal phase of the disease. | Cysts can appear in the feces from 3 days to 3 weeks after infection, and usually begin around the onset of symptomes. Shedding can last for weeks after the symptoms resolve. | Is fecal-oral through the ingestion of viable cysts of animal (animal-to-human) or human origin (person-to-person). Transmission occurs via direct contact or through contaminated food and water. Sexual behaviours such as oral-anal, genital-anal, and digital anal contact, can also facilitate transmission amongst humans. | Outbreaks of Giardiasis peak during the late summer and fall months | Worldwide distribution. The pathogen infects roughly 2% of adults and 6 to 8% of children in developed countries and 20 to 60% of people (mainly children) in developing countries. | 2*, 3, 8* | Some circulation is possible. In 2008, the prevelence of Giardia in France was estimated at 1.9%. A 1990 study also estimated that 1 to 4 % of children in day-care centers in Paris were carriers of the pathogen. In 2008 and 2009, 393 and 442 cases of giardia infections in humans were reported in the country respectively. | Humans and mammals such as primates, dogs, cats, cattle, sheep, pigs, rodents, beavers, and bears | *[141,142]* | *[2,147–153]* |
| | | | Assemblage B | | | | | | | | | | | | |
| | **Airborne diseases** | | | | | | | | | | | | | | |
| 26 | *Influenza* | *Influenza A virus* | | Yes | 1 to 4 days, usually 2 days | Communicability is correlated with viral shedding which typically begins 1 day prior to the onset of symptoms and lasts 3 to 5 days in adults and 7 to 10 days in children. | Studies have found influenza RNA in the stool of patients with seasonal influenza infection, several citing detection in the order of 30 to 40% of cases. The duration of fecal shedding however, remains unclear. | Airborn tramission occurs via droplets and small particle aerosols spread through coughing, sneezing, talking and breathing. Direct and indirect contact transmission also contributes to disease spread through coughing or sneezing for example. | In temperate climates, epidemics occur primarily during winter months. In tropical areas, the seasonal pattern is less pronounced, with cases occuring throughout the year. | Worldwide distribution. Influenza causes roughly 1 billion infections, 3 to 5 million cases of severe illness and 290,000 to 650,000 deaths every year across the globe. | 6, 7, 8** | Some circulation is possible. In metropolitan France, epidemics occur yearly between November and April. | Large host range including : humans, swine, horses, marine mammals, and birds | *[44,46,154–157]* | *[2,158–165]* |
| 27 | | *Influenza B virus* | | | | | | | | | | | Humans and seals | | |
| 28 | *MERS-CoV* | *MERS-CoV* | | No evidence | | | | | | | | | | | |

| | | | | | | | | | | | | | | | |
|---|---|---|---|---|---|---|---|---|---|---|---|---|---|---|---|
| 29 | Tuberculosis | *Mycobacterium tuberculosis complex (including Mycobacterium tuberculosis, M. bovis, M. africanum, M. pinnipedii, M. microti, M. caprae and M. canettii)* | | Yes | Usually 2 to 10 weeks<br><br>For latent TB infections, 5% of patients develop an active infection within 2 years and 5% develop an infection within their lifetime | Is highly variable, but in theory, infected persons can transmit the pathogen for as long as viable tubercle bacilli are discharged in the sputum. For smear positive or symptomatic infections, it may begin up to 3 months prior to respiratory symptom onset, where in smear negative asymptomatic cases, it can start 4 weeks prior to diagnosis. | | Primarily person-to-person and occurs most commonly via inhalation of aerosolized droplet nuclei spread through coughing (airborne transmission). Transmission can also occur via direct invasion through mucous membranes (ex: venereal transmission) or breaks in the skin (percutaneous transmission). Animal-to-person transmission though rare, has also been recorded through aerosols, fomites and animal bites. Moreover, bovine tuberculosis can result from exposure to infected cattle. In these cases, infection occurs through airborne spread or ingestion of contaminated milk or dairy products. | Studies suggest a seasonal pattern of tuberculosis with an increase in cases during spring and summer months in most countries. This points to an increase in the risk of transmission during the winter season. | Worldwide distribution, all countries are affected. In 2021, 10.6 million incidences and 1.6 million deaths were reported from the disease. The highest number of cases occurred in the South-East Asian Region (46%), followed by the African Region (23%) and the Western Pacific (18%). Tuberculosis is the 2nd largest killer amongst infectious diseases and 13th overall leading cause of death in the world. Roughly 1/4 of the world's population is believed to be infected with the pathogen. | 6 | Some circulation is possible. In France, as in most Western European countries, the disease is infrequent. Its incidence has been under 10 cases per 100,000 inhabitants/year for the last 10 years, and appears to be decreasing. However, in 2020, Île-de-France was the third region with the highest rate of TB (following French Guiana and Mayotte), with 14.3 cases per 100,000 inhabitants. Seine-Saint Denis is the department most affected, with 23.8 cases per 100,000 inhabitants. | Monkeys, humans, parrots, cattle, sheep, goats, dogs, cats | *[27,166]* | *[2,99,166–173]* |
| 4 | *Alphacoronavirus* | *HCoV-229E* | | Yes | 2 to 5 days | The specific period of communicability for this virus is not well understood | Studies have found *HCoV-229E* and *HCoV-NL63* in stool samples, but the exact duration of viral shedding remains unknown. | Person-to-person transmission via respiratory droplets generated through coughing, sneezing, talking or breathing | In temperate climates, cases of *HCoV-229E* and *HCoV-NL63* infections tend to increase in winter months and in the early spring. In Hong Kong *cases* seem to peak during the spring and summer months, indicating seasonality may not be limited to the winter in tropical and subtropical countries. | Worldwide distribution. | 6, 7, 8** | Some circulation is possible. HCoV-229E and HCoV-NL63 are common virus, and along with HCoV-OC43 and HCoV-HKU1 are believed to contribute to approximately 1/3 of all common cold infections in humans. Studies have shown the viruses are in circulation in France. | Humans, bats, camelids | *[46]* | *[174–185]* |
| | | *HCoV-NL63* | | No evidence | 2 to 4 days | | | | | | | | Humans, bats, palm civets | | |
| 5 | *Betacoronavirus* | *Betacoronavirus 1* | *HCoV-OC43* | Yes | 2 to 4 days | The specific period of communicability for this virus is not well understood. | Studies have found *HCoV-OC43* in stool samples, but the exact duration of viral shedding remains unknown. | Person-to-person transmission via respiratory droplets spread through coughing, sneezing, talking or breathing | In temperate climates, *HCoV-OC43* exhibits seasonality, peaks of cases in the winter and early spring. | Worldwide distribution. | 6, 7, 8** | Some circulation is possible. Studies have shown the virus is in circulation in France. Together, HCoV-229E, HCoV-NL63, HCoV-HKU1 and HCoV-OC43 are believed to contribute to approximately 1/3 of common cold infections in humans. | Humans, cows, horses, dogs, camels, deer, antelope and giraffes | *[46]* | *[176,177,179,180,182–185]* |
| | | *HCoV-HKU1* | | Yes | 2 to 4 days | The specific period of communicability for this virus is not well understood. | Studies have found HCoV-HKU1 in stool samples, but the exact duration of viral shedding remains unknown. | | In temperate climates, *HCoV-HKU1* exhibits seasonality, peaks of cases in the winter and early spring. | Worldwide distribution. | 6, 7, 8** | | Humans, mice | *[186]* | *[177,180,183,185]* |
| | **Emerging and vector- borne diseases** | | | | | | | | | | | | | | |
| 30 | Dengue | *Dengue virus (DENV)* | | No evidence | | | | | | | | | | | |
| 31 | Yellow fever | *Yellow fever virus (YFV)* | | No evidence | | | | | | | | | | | |
| 32 | Chikungunya | *Chikungunya virus (CHIKV)* | | No evidence | | | | | | | | | | | |
| 33 | Zika | *Zika virus (ZIKV)* | | No evidence | | | | | | | | | | | |
| 34 | West Nile | *West Nile virus (WNV)* | | No evidence | | | | | | | | | | | |
| 35 | Ebola | *Zaire ebolavirus (EBOV)* | | Yes | 2 to 21 days, usually 4 to 10 days | Infected persons can transmit the disease from the onset of symptoms and for as long as blood and body fluids secretions contain the virus, including the post-mortem period. | Ebola virus RNA has been found in stool for 29 days and in urine for 64 days after the onset of illness | Person-to-person transmission can occur by direct contact with blood, body fluids, tissues or semen of an infected person via broken skin or mucous membranes (ex: through unprotected sexual contact). Transmission can also occur through indirect contact with contaminated objects (formites).<br><br>Animal-to-human transmission is also possible via close contact with infected animals (alive or dead) or their body fluids. | Possible seasonality. A study found higher spillover intensity during periods of transitions between wet and dry seasons. | Since it was first identified in 1976, outbreaks of Ebola virus disease (EVD) cases have occured mainly in areas surrounding rain forests in the Democratic Republic of Congo (DRC), Gabon, the Republic of the Congo, South Sudan and Uganda. In 2014, an outbreak occured for the first time in West Africa (Guinea, Liberia and Sierra Leone), which saw large scale transmission in urban areas. During this epidemic, imported cases were reported in multiple countries, notably : Italy, Mali, Nigeria, Senegal, Spain, the UK and the US. | 2 | None. No case of Ebola has ever been reported in France.While sporadic imported cases are possible, according to the World Health Organization, the risk of Ebola spreading in Europe is very low. | Humans, fruit bats (Pteropodidae) and various monkey species (chimpanzees, gorillas, baboons, and duikers) | *[187]* | *[2,188–198]* |
| | | *Sudan ebolavirus (SUDV)* | | | | | | | | | | | | | |
| | | *Tai Forest ebolavirus (TAFV)* | | | | | | | | | | | | | |
| | | *Bundibugyo ebolavirus (BDBV)* | | | | | | | | | | | | | |

| | | | | | | | | | | | | | | | |
|---|---|---|---|---|---|---|---|---|---|---|---|---|---|---|---|
| 36 | Marburg virus disease | *Marburg virus (MARV)* | | No evidence | | | | | | | | | | | |
| 37 | Tularemia | *Francisella tularensis* | | No evidence | | | | | | | | | | | |
| 38 | Typhus | *Rickettsia prowazekii* | | No evidence | | | | | | | | | | | |
| | | *Rickettsia typhi* | | | | | | | | | | | | | |
| 39 | | *Orientia tsutsugamushi* | | | | | | | | | | | | | |
| 40 | Malaria | *Plasmodium falciparum* | | No evidence | | | | | | | | | | | |
| | | *Plasmodium knowlesi* | | | | | | | | | | | | | |
| | | *Plasmodium malariae* | | | | | | | | | | | | | |
| | | *Plasmodium ovale* | | | | | | | | | | | | | |
| | | *Plasmodium vivax* | | | | | | | | | | | | | |
| 41 | Arenavirus diseases (Lassa, Junin, Machupo, Guanarito, Sabiá) | | | No evidence | | | | | | | | | | | |
| 42 | Plague | *Yersinia pestis* | | No evidence | | | | | | | | | | | |
| 43 | Severe acute respiratory syndrome (SARS) | *Severe acute respiratory syndrome–related coronavirus (SARSr-CoV)* | Severe acute respiratory syndrome coronavirus 1 (SARS-CoV-1) | Yes* | 2 to 10 days (5 days on average), with isolated cases of longer incubation periods extending to 16 days | Not well understood, but appears to begin after the onset of symptoms and to persist for roughly 21 days | Viral shedding in feces begins after the onset of symptoms and can persist for over 23 days | Person-to-person transmission by direct mucous membrane contact with body fluids and/or respiratory droplets of an infected person. Transmission can also be indirect through contact with contaminated fomites. Other possible modes of spred include through inhalation of infectious aerosols, blood transfusions, or via sharps injuries. | | SARS was first reported in Asia in February 2003. In the months that followed, the illness spread to 29 countries in North America, South America, Europe, and Asia before the global outbreak was contained. No additional case has been reported since 2004. | 6, 7 | None. From November 2002 to July 2003, 7 cases of SARS were reported in France. However, no known human cases have been reported anywhere in the world since 2004. | Humans, Himalayan palm civets, racoon dogs, Chinese ferret badgers, cats, and pigs | *[199]* * | *[2,200–206]* |
| 44 | Smallpox | *Variola virus* | Variola major | No evidence | | | | | | | | | | | |
| | | | Variola minor | | | | | | | | | | | | |
| 45 | Crimean-Congo haemorrhagic fever | *Crimean-Congo hemorrhagic fever orthonairovirus (CCHFV)* | | No evidence | | | | | | | | | | | |
| 46 | Rift Valley fever | *Rift Valley fever virus (RVFV)* | | No evidence | | | | | | | | | | | |
| 47 | Tick-borne encephalitis | *Tick-borne encephalitis virus (TBEV)* | | No evidence | | | | | | | | | | | |
| 48 | Mpox | *Monkeypox virus (MPXV)* | | Yes | 7 to 17 days | It appears an infected person can transmit the disease from the time of symptom onset until the rash has fully healed. New data however suggests the communicability period may begin 1 to 4 days prior to illness onset in certain cases. | | Animal-to-human transmission occurs through direct contact with the blood, body fluids, or lesions of an infected animal. Likewise, person-to-person transmission can transpire through direct contact with the skin lesions/scabs, body fluids or mucosal surfaces of an infected person. Spread may also occur via contact with infectious respiratory droplets generated through talking, breathing, coughing, and sneezing. Vertical transmission and indirect transmission through contact with infected formites, have also been reported. | Mpox has no identifiable seasonality. | Previously, cases of mpox were mostly limited to endemic countries in West and Central Africa. Since early May 2022 cases have been and continue to be reported worldwide in over 100 endemic and non-endemic and countries, including France. | 2 | Some circulation is possible. On january 24th 2023, 4 982 cases (of which 4 128 were confirmed) had been reported in France. The region most affected was Ile-de-France with 3 119 cases. | Humans, squirrels, non-human primates, black-tailed prairie dogs, African brush-tailed porcupines, rats, and shrews | *[207–209]* | *[210–214]* |

| | | | | | | | | | | | | | | | |
|---|---|---|---|---|---|---|---|---|---|---|---|---|---|---|---|
| 49 | Coronavirus disease (COVID-19) | *Severe acute respiratory syndrome–related coronavirus (SARSr-CoV)* | Severe acute respiratory syndrome coronavirus 2 (SARS-CoV-2) | Yes | 2 to 14 days (median of 5 to 6 days) | Varies between virus variants, but typically an infected person can transmit the disease 2 days before the onset of symptoms. The period of communicability in adults with mild to moderate COVID-19 can persist for 10 days after symptoms begin. In severe to critically ill patients this period may extend to 20 days. | Data on viral shedding of SARS-CoV-2 in feces is variable in the literature. Prevalence of fecal RNA amongst COVID-19 patients appears to be around the order of 35 to 60%. The duration of pathogen shedding is believed to persists from 20 to 33 days on average, depending on the study. Further, shedding may be longer (28 to 42 days) among children. | Person-to-person transmission occurs via direct contact with secretions from the nose or mouth of an infected person. This occurs primarily through infectious droplets or aerosols generated through coughing, sneezing, talking or breathing. Indirect tranmission may also occur via contact with infected formites. | There is no clear consensus on SARS-CoV-2 seasonality. However, deaths attributed to the virus have been shown to decrease during the summer period in temperate regions, which would be consistent with the winter seasonality exihited by other human coronaviruses such as 229E, HKU1, NL63, and OC43. | Worldwide distribution, nearly all countries are affected. | 4, 6 | Some circulation is likely | The virus has a wide range of hosts including humans, non-human primates, mink, lions, tigers, deer, hamsters, cats, dogs, ferrets | *[46,155,156]* | *[215–224]* |
| | **Zoonoses** | | | | | | | | | | | | | | |
| 50 | Leptospirosis | 64 species have been identified | | Yes | 2 to 30 days, usually 5 to 14 days | Direct person-to-person transmission is rare, although, in theory, infected persons can spread the disease for as long bacteria is shed in their urine | Leptospires are usually excreted for 1 month, however cases of shedding lasting up to 11 months after acute illness have been reported | Transmission occurs though contact of the skin or mucous membranes either directly with an infected animal or indirectly contact via soil, vegetation or water contaminated with urine from an infected animal. Infections can also ensue from ingestion of contaminated food/water or by inhalation of infectious droplet aerosols. Person-to-person transmission can occur, but is rare. | In tropical countries, cases of the disease peak during the rainy season, where in temperate countries incidence increase during summer and autumn months. | Worldwide distribution with the exception of polar regions. Incidence is 50 to 100 times higher in tropical regions. | 1 | Some circulation is possible. Leptospirosis is endemic in many French overseas territorie, notably in: Martinique, Guadeloupe, French Guyana, Reunion Island, New Caledonia, Mayotte and French Polynesia. In metropolitan France, 708 cases were reported in 2021. An incidence of 1,05 cases per 100 000 inhabitants was reported in the Île-de-France region in 2020 and 2021. | Pathogenic leptospires have a wide range of hosts including: rats, field mice, voles, shrews, hedgehogs, swine, cattle, dogs, horses, racoons, deer, squirrels, foxes, skunks, reptiles, amphibians and humans | *[225]* | *[2,226–229]* |
| 51 | Anthrax | *Bacillus anthracis* | | No evidence | | | | | | | | | | | |
| 52 | Rabies | *Rabies virus (RABV)* | | No evidence | | | | | | | | | | | |
| 53 | Nipah and henipaviral diseases | *Nipah virus* | | No evidence | | | | | | | | | | | |
| 54 | | *Hendra virus* | | | | | | | | | | | | | |
| 55 | Venezuelan equine encephalitis | *Venezuelan equine encephalitis virus* | | No evidence | | | | | | | | | | | |
| 56 | Glanders | *Burkholderia mallei* | | No evidence | | | | | | | | | | | |
| 57 | Echinococcosis | *E. multilocularis* | | Yes | 5 to 15 years | The cestode is not directly transmitted from person-to-person | | Transmission occurs almost exclusively via the oral route through the ingestion of viable eggs. Infection can occur via direct contact with contaminated hosts or via indirect contact with environemental formites or consumption of contaminated food/water. | | Northern Hemisphere: central and northern Europe, Central Asia, northern Russia, northern Japan, north-central United States, northwestern Alaska, and northwestern Canada | 1 | Some circulation is possible. Human infections remain rare in europe, with an annual incidence of 0.74 per 100,000 inhabitants. In France, roughly 30 cases are reported every year, mainly in Franche-Comté, Bourgogne, Rhône Alpes and Auvergne (endemic area). | Definite hosts : mainly foxes, but other canids such as wolves, coyotes, dogs and cats have been infected Intermediate hosts : voles, lemmings, shrews and mice *Humans are accidental intermediate hosts | *[230]* | *[231–234]* |
| 58 | | *E. granulosus* | | No evidence | | | | | | | | | | | |
| | **Sexually transmitted infections** | | | | | | | | | | | | | | |
| 59 | Hepatitis B | *Hepatitis B virus (HBV)* | | No evidence | | | | | | | | | | | |
| 60 | Human immunodeficiency virus (HIV) infection | *Human immunodeficiency virus 1 (HIV-1)*; *Human immunodeficiency virus 2 (HIV-2)* | | Yes | Usually 1 to 3 months | The exact period is unknown, but believed to beging not long after infection and extend throughout the life of the infected individual | | Person-to-person transmission occurs via the exchange of biological fluids such as blood, breast milk, semen and vaginal secretions from an infected person. This can occur through sexual activity, high-risk injections (ex: intravenous drugs), transfussions and from mother to child (vertical transmission). Nonetheless, sexual transmission accounts for over 90% of infections worldwide. | No clear seasonality, cases occur around the world year-round | Worldwide distribution. In 2021, 0.7% of adults aged 15 to 49 and 38.4 million people globally were living with HIV. 95% of HIV infections occur in developpong countries. Today, the most affected area in the world is Sub-Saharan Africa. | 2 | Some circulation is expected. Every year 6600 people find out that they are HIV seropositive in France. In 2021, 0.3% of adults aged 15 to 49 and 190 000 people were living with HIV in the country. The department most affected is French Guiana, with a prevelence of HIV that exceeds 1%. | Humans | *[235]* ** | *[236–241]* |

**Note: Studies that dealt with spiked wastewater samples or that described open sewage systems, were not considered when evaluating detectability in wastewater**

***Paper deals with hospital wastewater**

****Paper is a preprint**

***Legend:***

■ = **Pathogens identified as being of interest for general public health surveillance for the 2024 OPG based on a risk map produced by Santé publique France for the event and a literature search which aimed to identify infectious diseases of concern for mass gatherings**
■ = **Pathogens meeting the analytical feasibility criterion, proposed by the expert panel for consideration**

**1 = Pathogen/disease not subject to specific surveillance in France**
**2 = Nationally notifiable diseases in France** — https://www.santepubliquefrance.fr/maladies-a-declaration-obligatoire
**2* = Nationally notifiable disease due to its classification as a foodborne hazard capable of causing outbreaks of foodborne illness**

**3 = Sentinel Network for Acute Diarrhea (*weekly surveillance*)** — https://www.sentiweb.fr/france/fr/?page=maladies&mal=6
**4 = National wastewater monitoring system for SARS-CoV-2 in France (SUM'Eau)** — https://sante.gouv.fr/archives/archives-presse/archives-communiques-de-presse/article/le-ministere-des-solidarites-et-de-la-sante-et-le-ministere-de-la-transition
**5 = Enterovirus Surveillance Network (RSE), coordinated by the National Reference Center (CNR) and Santé publique France (*monthly surveillance*)** — https://www.santepubliquefrance.fr/content/download/357479/3085298
**6 = Sentinel Network for Acute Respiratory Infections (*weekly surveillance*)** — https://www.sentiweb.fr/france/fr/?page=maladies&mal=25
**7 = Sentinel Network for Flu-like Illnesses (*weekly surveillance*)** — https://www.sentiweb.fr/france/fr/?page=maladies&mal=3
***Note : Since 2022, the incidences of this indicator have been estimated retrospectively based on the Acute Respiratory Infections indicator**

**Surveillance of Emergency Departments and Deaths (SurSaUD®):** — https://www.santepubliquefrance.fr/surveillance-syndromique-sursaud-R
**8 = *SOS Médecins* Network** — https://www.santepubliquefrance.fr/surveillance-syndromique-sursaud-R/reseau-sos-medecins
**8* = syndromic grouping for acute gastroenteritis**
**8** = syndromic grouping for influenza**

**Note : surveillance systems that report annually were not considered given the durations the 2024 Paris Olympic (17 days: July 26 to August 11) and Paralympic Games (12 days: August 28 to September 8)**

**Sources**


[1] Benschop KSM, van der Avoort HG, Jusic E, Vennema H, van Binnendijk R, Duizer E. Polio and Measles Down the Drain: Environmental Enterovirus Surveillance in the Netherlands, 2005 to 2015. Applied and Environmental Microbiology 2017;83:e00558-17. https://doi.org/10.1128/AEM.00558-17.
[2] Heymann DL. Control of communicable diseases manual. American Public Health Association; 2008.
[3] World Health Organization. Measles. World Health Organization 2019.
[4] Permar SR, Moss WJ, Ryon JJ, Monze M, Cutts F, Quinn TC, et al. Prolonged Measles Virus Shedding in Human Immunodeficiency Virus—Infected Children, Detected by Reverse Transcriptase—Polymerase Chain Reaction. The Journal of Infectious Diseases 2001;183:532–8.
[5] Public Health Agency of Canada. Pathogen Safety Data Sheets: Infectious Substances – Measles virus. Government of Canada 2011.
[6] World Health Organization. A 30-fold rise of measles cases in 2023 in the WHO European Region warrants urgent action 2023.
[7] Assurance Maladie France. Vaccins obligatoires. l'Assurance Maladie 2022.
[8] Hovi T, Stenvik M, Partanen H, Kangas A. Poliovirus surveillance by examining sewage specimens. Quantitative recovery of virus after introduction into sewerage at remote upstream location. Epidemiol Infect 2001;127:101–6.
[9] Hovi T, Shulman LM, Avoort HVD, Deshpande J, Roivainen M, Gourville EMD. Role of environmental poliovirus surveillance in global polio eradication and beyond. Epidemiology & Infection 2012;140:1–13. https://doi.org/10.1017/S095026881000316X.
[10] World Health Organization. Poliomyelitis. World Health Organization 2022.
[11] World Health Organization. Poliomyelitis (polio) n.d.
[12] Institut Pasteur. Poliomyélite. Institut Pasteur 2021.
[13] World Health Organization. Detection of circulating vaccine derived polio virus 2 (cVDPV2) in environmental samples– the United Kingdom of Great Britain and Northern Ireland and the United States of America. World Health Organization 2022.
[14] Malvy DJ, Drucker J. Elimination of poliomyelitis in France: epidemiology and vaccine status. Public Health Rev 1993;21:41–9.
[15] Polio Global Eradication Initiative. GPEI-Vaccine-Derived Polioviruses n.d.
[16] Martinez-Bakker M, King AA, Rohani P. Unraveling the Transmission Ecology of Polio. PLoS Biol 2015;13:e1002172. https://doi.org/10.1371/journal.pbio.1002172.
[17] Centers for Disease Control and Prevention. Polio: For Healthcare Providers. Centers for Disease Control and Prevention 2022.
[18] Mehndiratta MM, Mehndiratta P, Pande R. Poliomyelitis. Neurohospitalist 2014;4:223–9. https://doi.org/10.1177/1941874414533352.
[19] Redha MA, Al Sweih N, Albert MJ. Virulence and phylogenetic groups of Escherichia coli cultured from raw sewage in Kuwait. Gut Pathogens 2022;14:18. https://doi.org/10.1186/s13099-022-00490-4.
[20] European Centre for Disease Prevention and Control. STEC infection - Annual Epidemiological Report for 2021. Stockholm: European Centre for Disease Prevention and Control; 2022.
[21] Public Health Agency of Canada. Pathogen Safety Data Sheets: Infectious Substances – Escherichia coli, enterohemorrhagic. Government of Canada 2015.
[22] Ministry of Health New Zealand. Verocytotoxin- or Shiga toxin-producing Escherichia coli (VTEC/STEC). New Zealand Government 2021.
[23] World Health Organization. E. coli. World Health Organization 2018.
[24] Zohra T, Ikram A, Salman M, Amir A, Saeed A, Ashraf Z, et al. Wastewater based environmental surveillance of toxigenic Vibrio cholerae in Pakistan. PLOS ONE 2021;16:e0257414. https://doi.org/10.1371/journal.pone.0257414.
[25] Madico G, Checkley W, Gilman R, Bravo N, cabrera rojo Iilia zulema, Calderon M, et al. Active Surveillance forVibrio choleraeO1 and Vibriophages in Sewage Water as a Potential Tool To Predict Cholera Outbreaks. Journal of Clinical Microbiology 1996;34:2968–72. https://doi.org/10.1128/JCM.34.12.2968-2972.1996.
[26] Teklehaimanot GZ, Genthe B, Kamika I, Momba MNB. Prevalence of enteropathogenic bacteria in treated effluents and receiving water bodies and their potential health risks. Science of The Total Environment 2015;518–519:441–9. https://doi.org/10.1016/j.scitotenv.2015.03.019.
[27] Cai L, Zhang T. Detecting Human Bacterial Pathogens in Wastewater Treatment Plants by a High-Throughput Shotgun Sequencing Technique. Environ Sci Technol 2013;47:5433–41. https://doi.org/10.1021/es400275r.
[28] World Health Organization. Cholera. World Health Organization 2022.
[29] Public Health Agency ofCanada. Pathogen Safety Data Sheets: Infectious Substances – Vibrio cholerae. Government of Canada 2011.
[30] Centers for Disease Control and Prevention. Cholera. Centers for Disease Control and Prevention 2020.
[31] Santé Publique France. Choléra. Santé Publique France 2022.
[32] Usmani M, Brumfield KD, Magers BM, Chaves-Gonzalez J, Ticehurst H, Barciela R, et al. Combating cholera by building predictive capabilities for pathogenic Vibrio cholerae in Yemen. Sci Rep 2023;13:2255. https://doi.org/10.1038/s41598-022-22946-y.
[33] Yan T, O'Brien P, Shelton JM, Whelen AC, Pagaling E. Municipal Wastewater as a Microbial Surveillance Platform for Enteric Diseases: A Case Study for Salmonella and Salmonellosis. Environ Sci Technol 2018;52:4869–77. https://doi.org/10.1021/acs.est.8b00163.
[34] Public Health Agency of Canada. Pathogen Safety Data Sheets: Infectious Substances – Salmonella enterica spp. Government of Canada 2011.
[35] Institut Pasteur. Rapports d'activité du CNR des Escherichia coli, Shigella, Salmonella. Institut Pasteur 2020.
[36] Ravel A, Smolina E, Sargeant JM, Cook A, Marshall B, Fleury MD, et al. Seasonality in Human Salmonellosis: Assessment of Human Activities and Chicken Contamination as Driving Factors. Foodborne Pathogens and Disease 2010;7:785–94. https://doi.org/10.1089/fpd.2009.0460.
[37] Centers for Disease Control and Prevention. Salmonellosis (Nontyphoidal) - Chapter 4 - 2020 Yellow Book | Travelers' Health | CDC. Centers for Disease Control and Prevention 2019.
[38] World Health Organization. Salmonella (non-typhoidal). World Health Organization 2018.
[39] Ministère de l'Agriculture et de la Souveraineté alimentaire. La salmonellose non typhique. Ministère de l'Agriculture et de la Souveraineté alimentaire 2017.
[40] Institut Pasteur. Salmonella infection. Institut Pasteur 2021.
[41] Hellmér M, Paxéus N, Magnius L, Enache L, Arnholm B, Johansson A, et al. Detection of Pathogenic Viruses in Sewage Provided Early Warnings of Hepatitis A Virus and Norovirus Outbreaks. Applied and Environmental Microbiology 2014;80:6771–81. https://doi.org/10.1128/AEM.01981-14.

[42] La Rosa G, Iaconelli M, Pourshaban M, Muscillo M. Detection and molecular characterization of noroviruses from five sewage treatment plants in central Italy. Water Research 2010;44:1777–84. https://doi.org/10.1016/j.watres.2009.11.055.
[43] Mabasa VV, Meno KD, Taylor MB, Mans J. Environmental Surveillance for Noroviruses in Selected South African Wastewaters 2015–2016: Emergence of the Novel GII.17. Food Environ Virol 2018;10:16–28. https://doi.org/10.1007/s12560-017-9316-2.
[44] Markt R, Stillebacher F, Nägele F, Kammerer A, Peer N, Payr M, et al. Expanding the Pathogen Panel in Wastewater Epidemiology to Influenza and Norovirus. Viruses 2023;15:263. https://doi.org/10.3390/v15020263.
[45] Symonds EM, Griffin DW, Breitbart M. Eukaryotic Viruses in Wastewater Samples from the United States. Appl Environ Microbiol 2009;75:1402–9. https://doi.org/10.1128/AEM.01899-08.
[46] Rothman JA, Loveless TB, Kapcia J, Adams ED, Steele JA, Zimmer-Faust AG, et al. RNA Viromics of Southern California Wastewater and Detection of SARS-CoV-2 Single-Nucleotide Variants. Applied and Environmental Microbiology 2021;87:e01448-21. https://doi.org/10.1128/AEM.01448-21.
[47] Centers for Disease Control and Prevention. How Norovirus Spreads. Centers for Disease Control and Prevention 2021.
[48] Public Health Agency of Canada. Pathogen Safety Data Sheets: Infectious Substances – Norovirus. Government of Canada 2020.
[49] Wu Q, Xuan Z, Liu J, Zhao X, Chen Y, Wang C, et al. Norovirus shedding among symptomatic and asymptomatic employees in outbreak settings in Shanghai, China. BMC Infectious Diseases 2019;19:592. https://doi.org/10.1186/s12879-019-4205-y.
[50] Government of Canada. Norovirus: For health professionals 2022.
[51] World Health Organization. Foodborne viral disease in the European region. World Health Organization 2015.
[52] Anses. Comment éviter les intoxications alimentaires liées aux norovirus ? Anses - Agence nationale de sécurité sanitaire de l'alimentation, de l'environnement et du travail 2023.
[53] O'Brien E, Nakyazze J, Wu H, Kiwanuka N, Cunningham W, Kaneene JB, et al. Viral diversity and abundance in polluted waters in Kampala, Uganda. Water Research 2017;127:41–9. https://doi.org/10.1016/j.watres.2017.09.063.
[54] Barril PA, Fumian TM, Prez VE, Gil PI, Martínez LC, Giordano MO, et al. Rotavirus seasonality in urban sewage from Argentina: Effect of meteorological variables on the viral load and the genetic diversity. Environmental Research 2015;138:409–15. https://doi.org/10.1016/j.envres.2015.03.004.
[55] Dubois E, Le Guyader F, Haugarreau L, Kopecka H, Cormier M, Pommepuy M. Molecular epidemiological survey of rotaviruses in sewage by reverse transcriptase seminested PCR and restriction fragment length polymorphism assay. Applied and Environmental Microbiology 1997;63:1794–800. https://doi.org/10.1128/aem.63.5.1794-1800.1997.
[56] Cortese M, Haber P. Rotavirus. Centers for Disease Control and Prevention 2022.
[57] Burke RM, Tate JE, Kirkwood CD, Steele AD, Parashar UD. Current and new rotavirus vaccines. Current Opinion in Infectious Diseases 2019;32:435.
[58] European Centre for Disease Prevention and Control. Disease factsheet about rotavirus. European Centre for Disease Prevention and Control n.d.
[59] Government of New South Wales. Rotavirus infection fact sheet - Fact sheets. NSW Government 2012.
[60] Public Health Agency of Canada. Rotavirus vaccine: Canadian Immunization Guide. Government of Canada 2021.
[61] Kumar D, Shepherd FK, Springer NL, Mwangi W, Marthaler DG. Rotavirus Infection in Swine: Genotypic Diversity, Immune Responses, and Role of Gut Microbiome in Rotavirus Immunity. Pathogens 2022;11:1078. https://doi.org/10.3390/pathogens11101078.
[62] World Health Organization. Immunization coverage 2022.
[63] Ministère de la santé et de la prévention. Gastroentérites aigües à rotavirus. Ministère de la Santé et de la Prévention 2022.
[64] Iaconelli M, Valdazo-González B, Equestre M, Ciccaglione AR, Marcantonio C, Della Libera S, et al. Molecular characterization of human adenoviruses in urban wastewaters using next generation and Sanger sequencing. Water Research 2017;121:240–7. https://doi.org/10.1016/j.watres.2017.05.039.
[65] Reyne MI, Allen DM, Levickas A, Allingham P, Lock J, Fitzgerald A, et al. Detection of human adenovirus F41 in wastewater and its relationship to clinical cases of acute hepatitis of unknown aetiology. Science of The Total Environment 2023;857:159579. https://doi.org/10.1016/j.scitotenv.2022.159579.
[66] Lynch JP, Kajon AE. Adenovirus: Epidemiology, Global Spread of Novel Serotypes, and Advances in Treatment and Prevention. Semin Respir Crit Care Med 2016;37:586–602. https://doi.org/10.1055/s-0036-1584923.
[67] Lee B, Damon CF, Platts-Mills JA. Pediatric acute gastroenteritis due to adenovirus 40/41 in low- and middle-income countries. Curr Opin Infect Dis 2020;33:398–403. https://doi.org/10.1097/QCO.0000000000000663.
[68] D'Souza DH. 5 - Update on foodborne viruses: types, concentration and sampling methods. In: Sofos J, editor. Advances in Microbial Food Safety, Oxford: Woodhead Publishing; 2015, p. 102–16. https://doi.org/10.1533/9781782421153.1.102.
[69] Public Health Agency of Canada. Pathogen safety data sheet: Infectious substances – Adenovirus (serotypes 40 and 41). Government of Canada 2011.
[70] Pothier P, Bultel C, Ferre-Aubineau V, Gantzer C, Hartemann P, Legea O, et al. Bilan des connaissances relatives aux virus transmissibles à l'homme par voie orale 2007.
[71] Gerba CP. Chapter 22 - Environmentally Transmitted Pathogens. In: Pepper IL, Gerba CP, Gentry TJ, editors. Environmental Microbiology (Third Edition), San Diego: Academic Press; 2015, p. 509–50. https://doi.org/10.1016/B978-0-12-394626-3.00022-3.
[72] Haque E, Banik U, Monwar T, Anthony L, Adhikary AK. Worldwide increased prevalence of human adenovirus type 3 (HAdV-3) respiratory infections is well correlated with heterogeneous hypervariable regions (HVRs) of hexon. PLOS ONE 2018;13:e0194516. https://doi.org/10.1371/journal.pone.0194516.
[73] Zhang S, Shi J, Sharma E, Li X, Gao S, Zhou X, et al. In-sewer decay and partitioning of Campylobacter jejuni and Campylobacter coli and implications for their wastewater surveillance. Water Research 2023;233:119737. https://doi.org/10.1016/j.watres.2023.119737.
[74] World Health Organization. Campylobacter. World Health Organization 2020.
[75] Lake IR, Colón-González FJ, Takkinen J, Rossi M, Sudre B, Dias JG, et al. Exploring Campylobacter seasonality across Europe using The European Surveillance System (TESSy), 2008 to 2016. Eurosurveillance 2019;24:1800028. https://doi.org/10.2807/1560-7917.ES.2019.24.13.180028.
[76] Santé Publique France. Campylobacter 2022.
[77] Missouri Department of Health & Senior Services. Campylobacteriosis | Food Safety | Health & Senior Services. MOGov n.d.
[78] Public Health Agency of Canada. Pathogen Safety Data Sheets: Infectious Substances – Campylobacter coli. Government of Canada 2012.
[79] Public Health Agency of Canada. Pathogen Safety Data Sheets: Infectious Substances – Campylobacter jejuni. Government of Canada 2012.
[80] Anses. Campylobacter jejuni Campylobacter coli : Data sheet on foodborne biological hazards. Anses 2011.
[81] European Centre for Disease Prevention and Control. Shigellosis. European Centre for Disease Prevention and Control 2017.
[82] Public Health Agency of Canada. Pathogen Safety Data Sheets: Infectious Substances – Shigella spp. 2011.
[83] Centers for Disease Control and Prevention. Shigella – Shigellosis. Centers for Disease Control and Prevention 2023.
[84] Institut Pasteur. Shigellosis. Institut Pasteur 2022.
[85] Khouja LBA, Cama V, Xiao L. Parasitic contamination in wastewater and sludge samples in Tunisia using three different detection techniques. Parasitol Res 2010;107:109–16. https://doi.org/10.1007/s00436-010-1844-8.
[86] Ajonina C, Buzie C, Möller J, Otterpohl R. The detection of Entamoeba histolytica and Toxoplasma gondii in wastewater. J Toxicol Environ Health A 2018;81:1–5. https://doi.org/10.1080/15287394.2017.1392399.
[87] R. Saleh FE, A. Gad M, A. Ashour A, I. Soliman M, M. El-Senousy W, Z. Al-Herrawy A. Molecular detection of Entamoeba histolytica in fresh vegetables and irrigation. Egyptian Journal of Aquatic Biology and Fisheries 2019;22:551–61. https://doi.org/10.21608/ejabf.2019.24756.
[88] Jaran A. Prevalence and seasonal variation of human intestinal parasites in patients attending hospital with abdominal symptoms in northern Jordan. Eastern Mediterranean Health Journal = La Revue de Santé de La Méditerranée Orientale = Al-Majallah al-Ṣiḥḥīyah Li-Sharq al-Mutawassiṭ 2016;22:756.
[89] Public Health Agency of Canada. Pathogen Safety Data Sheets: Infectious Substances – Entamoeba histolytic. Government of Canada 2014.
[90] Institut Pasteur. Amoebiasis. Institut Pasteur 2015.
[91] Chou A, Austin RL. Entamoeba Histolytica. StatPearls, Treasure Island (FL): StatPearls Publishing; 2023.
[92] Anses. Entamoeba histolytica, E. histolytica/E. dispar. Anses 2020.
[93] La Rosa G, Iaconelli M, Mancini P, Bonanno Ferraro G, Veneri C, Bonadonna L, et al. First detection of SARS-CoV-2 in untreated wastewaters in Italy. Science of The Total Environment 2020;736:139652. https://doi.org/10.1016/j.scitotenv.2020.139652.
[94] Clemente-Casares P, Pina S, Buti M, Jardi R, Martín M, Bofill-Mas S, et al. Hepatitis E Virus Epidemiology in Industrialized Countries. Emerg Infect Dis 2003;9:449–54. https://doi.org/10.3201/eid0904.020351.
[95] Ministère chargé de la santé. Hépatite A. Vacccination info servive fr 2019.
[96] Public Health Agency of Canada. Pathogen Safety Data Sheets: Infectious Substances – Hepatitis A virus (HAV). Government of Canada 2011.
[97] World Health Organization. Hepatitis A. World Health Organization 2022.
[98] INRS. Hépatite A. Agent pathogène - Base de données EFICATT - INRS 2015.
[99] Fares A. Seasonality of Hepatitis: A Review Update. J Family Med Prim Care 2015;4:96–100. https://doi.org/10.4103/2249-4863.152263.
[100] Alfonsi V, Romanò L, Ciccaglione AR, Rosa GL, Bruni R, Zanetti A, et al. Hepatitis E in Italy: 5 years of national epidemiological, virological and environmental surveillance, 2012 to 2016. Eurosurveillance 2018;23:1700517. https://doi.org/10.2807/1560-7917.ES.2018.23.41.1700517.
[101] Martínez Wassaf MG, Pisano MB, Barril PA, Elbarcha OC, Pinto MA, Mendes de Oliveira J, et al. First detection of hepatitis E virus in Central Argentina: Environmental and serological survey. Journal of Clinical Virology 2014;61:334–9. https://doi.org/10.1016/j.jcv.2014.08.016.
[102] Facts about hepatitis E. European Centre for Disease Prevention and Control 2017.
[103] Mansuy JM, Gallian P, Dimeglio C, Saune K, Arnaud C, Pelletier B, et al. A nationwide survey of hepatitis E viral infection in French blood donors. Hepatology 2016;63:1145–54. https://doi.org/10.1002/hep.28436.
[104] Public Health Agency of Canada. Pathogen Safety Data Sheets: Infectious Substances – Hepatitis E virus. Government of Canada 2011.
[105] Lund V, Fonahn W, Pettersen JE, Caugant DA, Ask E, Nysaeter Å. Detection of Legionella by cultivation and quantitative real-time polymerase chain reaction in biological waste water treatment plants in Norway. Journal of Water and Health 2014;12:543–54. https://doi.org/10.2166/wh.2014.063.
[106] Caicedo C, Beutel S, Scheper T, Rosenwinkel KH, Nogueira R. Occurrence of Legionella in wastewater treatment plants linked to wastewater characteristics. Environ Sci Pollut Res 2016;23:16873–81. https://doi.org/10.1007/s11356-016-7090-6.
[107] Catalan V, Garcia F, Moreno C, Vila MJ, Apraiz D. Detection of Legionella pneumophila in wastewater by nested polymerase chain reaction. Research in Microbiology 1997;148:71–8. https://doi.org/10.1016/S0923-2508(97)81902-X.
[108] World Health Organization. Legionellosis. World Health Organization n.d.
[109] Winn WC. Legionella. In: Baron S, editor. Medical Microbiology. 4th ed., Galveston (TX): University of Texas Medical Branch at Galveston; 1996.
[110] Public Health Agency of Canada. Pathogen Safety Data Sheets: Infectious Substances – Legionella pneumophila 2011.
[111] Institut Pasteur. Legionellosis: a novel mechanism by which the bacterium Legionella pneumophila regulates the immune response of its host cells. Institut Pasteur 2022.
[112] Kohler RB, Winn WC, Wheat LJ. Onset and duration of urinary antigen excretion in Legionnaires disease. J Clin Microbiol 1984;20:605–7.

[113] Santé Publique France. Légionellose en France : données épidémiologiques 2020. Santé Publique France 2021.

[114] Diederen BMW. Legionella spp. and Legionnaires' disease. Journal of Infection 2008;56:1–12. https://doi.org/10.1016/j.jinf.2007.09.010.

[115] Roulová N, Moťková P, Brožková I, Brzezinska MS, Pejchalová M. Detection, characterization, and antimicrobial susceptibility of Yersinia enterocolitica in different types of wastewater in the Czech Republic. Journal of Applied Microbiology 2022;133:2255–66. https://doi.org/10.1111/jam.15786.

[116] Centers for Disease Control and Prevention. Questions and Answers | Yersinia | CDC. Centers for Disease Control and Prevention 2019.

[117] Public Health Agency of Canada. Pathogen Safety Data Sheets: Infectious Substances – Yersinia enterocolitica. Government of Canada 2012.

[118] Schaake J, Kronshage M, Uliczka F, Rohde M, Knuuti T, Strauch E, et al. Human and Animal Isolates of Yersinia enterocolitica Show Significant Serotype-Specific Colonization and Host-Specific Immune Defense Properties. Infect Immun 2013;81:4013–25. https://doi.org/10.1128/IAI.00572-13.

[119] Institut Pasteur. Les infections à Yersinia. Institut Pasteur 2022.

[120] Anses. Yersinia enterocolitica, Yersinia pseudotuberculosis : Fiche de description de danger biologique transmissible par les aliments. Anses 2017.

[121] Ministère de l'Agriculture et de la Souveraineté alimentaire. La yersiniose entérique. Ministère de l'Agriculture et de la Souveraineté alimentaire 2017.

[122] Rmadi Y, Elargoubi A, González-Sanz R, Mastouri M, Cabrerizo M, Aouni M. Molecular characterization of enterovirus detected in cerebrospinal fluid and wastewater samples in Monastir, Tunisia, 2014–2017. Virol J 2022;19:45. https://doi.org/10.1186/s12985-022-01770-w.

[123] Tao Z, Chen P, Cui N, Lin X, Ji F, Liu Y, et al. Detection of enteroviruses in urban sewage by next generation sequencing and its application in environmental surveillance. Science of The Total Environment 2020;728:138818. https://doi.org/10.1016/j.scitotenv.2020.138818.

[124] Huang R, Lin X, Chen P, Ji F, Liu Y, Wang S, et al. Detection and diversity of human enteroviruses from domestic sewage in Weishan Lake region, eastern China, 2018–2019. Journal of Applied Microbiology 2023;134:lxad028. https://doi.org/10.1093/jambio/lxad028.

[125] Majumdar M, Sharif S, Klapsa D, Wilton T, Alam MM, Fernandez-Garcia MD, et al. Environmental Surveillance Reveals Complex Enterovirus Circulation Patterns in Human Populations. Open Forum Infect Dis 2018;5:ofy250. https://doi.org/10.1093/ofid/ofy250.

[126] Lizasoain A, Mir D, Masachessi G, Farías A, Rodríguez-Osorio N, Victoria M, et al. Environmental Surveillance through Next-Generation Sequencing to Unveil the Diversity of Human Enteroviruses beyond the Reported Clinical Cases. Viruses 2021;13:120. https://doi.org/10.3390/v13010120.

[127] Kitakawa K, Kitamura K, Yoshida H. Monitoring Enteroviruses and SARS-CoV-2 in Wastewater Using the Polio Environmental Surveillance System in Japan. Applied and Environmental Microbiology 2023;89:e01853-22. https://doi.org/10.1128/aem.01853-22.

[128] Public Health Agency of Canada. Pathogen Safety Data Sheets: Infectious Substances – Coxsackievirus 2001.

[129] National Centre for Infectious Diseases. Hand, Foot and Mouth Disease - National Centre for Infectious Diseases. National Centre for Infectious Diseases n.d.

[130] Lanrewaju AA, Enitan-Folami AM, Sabiu S, Edokpayi JN, Swalaha FM. Global public health implications of human exposure to viral contaminated water. Front Microbiol 2022;13:981896. https://doi.org/10.3389/fmicb.2022.981896.

[131] Centre National de Référence des Entérovirus et Parechovirus. Point sur les infections à entérovirus au 14 juin 2022. Santé Publique France 2022.

[132] Public Health Agency of Canada. Pathogen Safety Data Sheets: Infectious Substances – Echovirus. Government of Canada 2014.

[133] Antona D, Lévêque N, Chomel JJ, Dubrou S, Lévy-Bruhl D, Lina B. Surveillance of enteroviruses in France, 2000-2004. Eur J Clin Microbiol Infect Dis 2007;26:403–12. https://doi.org/10.1007/s10096-007-0306-4.

[134] Tedcastle A, Wilton T, Pegg E, Klapsa D, Bujaki E, Mate R, et al. Detection of Enterovirus D68 in Wastewater Samples from the UK between July and November 2021. Viruses 2022;14:143. https://doi.org/10.3390/v14010143.

[135] Erster O, Bar-Or I, Levy V, Shatzman-Steuerman R, Sofer D, Weiss L, et al. Monitoring of Enterovirus D68 Outbreak in Israel by a Parallel Clinical and Wastewater Based Surveillance. Viruses 2022;14:1010. https://doi.org/10.3390/v14051010.

[136] Weil M, Mandelboim M, Mendelson E, Manor Y, Shulman L, Ram D, et al. Human enterovirus D68 in clinical and sewage samples in Israel. Journal of Clinical Virology 2017;86:52–5. https://doi.org/10.1016/j.jcv.2016.11.013.

[137] National Collaborating Center for Infcetious Diseases. EV-D68. National Collaborating Centre for Infectious Diseases 2015.

[138] Centers for Disease Control and Prevention. Enterovirus D68 (EV-D68) | CDC 2022.

[139] Fall A, Kenmoe S, Ebogo-Belobo JT, Mbaga DS, Bowo-Ngandji A, Foe-Essomba JR, et al. Global prevalence and case fatality rate of Enterovirus D68 infections, a systematic review and meta-analysis. PLoS Negl Trop Dis 2022;16:e0010073. https://doi.org/10.1371/journal.pntd.0010073.

[140] Ong KC, Wong KT. Understanding Enterovirus 71 Neuropathogenesis and Its Impact on Other Neurotropic Enteroviruses. Brain Pathol 2015;25:614–24. https://doi.org/10.1111/bpa.12279.

[141] European Centre for Disease Prevention and Control. Outbreak of enterovirus A71 with severe neurological symptoms among children in Catalonia, Spain. Stockholm: ECDC; 2016.

[142] Martins FDC, Ladeia WA, Toledo R dos S, Garcia JL, Navarro IT, Freire RL. Surveillance of Giardia and Cryptosporidiumin sewage from an urban area in Brazil. Rev Bras Parasitol Vet 2019;28:291–7. https://doi.org/10.1590/S1984-29612019037.

[143] Zahedi A, Monis P, Deere D, Ryan U. Wastewater-based epidemiology—surveillance and early detection of waterborne pathogens with a focus on SARS-CoV-2, Cryptosporidium and Giardia. Parasitol Res 2021;120:4167–88. https://doi.org/10.1007/s00436-020-07023-5.

[144] Pumipuntu N, Piratae S. Cryptosporidiosis: A zoonotic disease concern. Vet World 2018;11:681–6. https://doi.org/10.14202/vetworld.2018.681-686.

[145] Costa D, Razakandrainibe R, Basmaciyan L, Raibaut J, Delaunay P, Morio F, et al. A summary of cryptosporidiosis outbreaks reported in France and overseas departments, 2017–2020. Food and Waterborne Parasitology 2022;27:e00160. https://doi.org/10.1016/j.fawpar.2022.e00160.

[146] Public Health Agency of Canada. Cryptosporidiosis (Cryptosporidium): For health professionals 2019.

[147] Gerace E, Lo Presti VDM, Biondo C. Cryptosporidium Infection: Epidemiology, Pathogenesis, and Differential Diagnosis. Eur J Microbiol Immunol (Bp) 2019;9:119–23. https://doi.org/10.1556/1886.2019.00019.

[148] Centers for Disease Control and Prevention. Transmission | Giardia | Parasites | CDC. Centers for Disease Control and Prevention 2021.

[149] Public Health Agency of Canada. Pathogen Safety Data Sheets: Infectious Substances – Giardia lamblia. Government of Canada 2012.

[150] Centers for Disease Control and Prevention. CDC - DPDx - Giardiasis. Centers for Disease Control and Prevention 2017.

[151] Hooshyar H, Rostamkhani P, Arbabi M, Delavari M. Giardia lamblia infection: review of current diagnostic strategies. Gastroenterol Hepatol Bed Bench 2019;12:3–12.

[152] Virot D, Golliot F. Investigation de cas groupés de giardiose parmi les passagers et l'équipage d'une croisière. Canal du Rhône à Sète, 28 septembre-4 octobre 2008 2010.

[153] Anses. Giardia duodenalis. Data Sheet on Foodborne Biological Hazards / Giardia Duodenalis 2011.

[154] American Academy of Pediatrics. Giardiasis—Child Care and Schools. Pediatric Patient Education 2021. https://doi.org/10.1542/ppe_document052.

[155] Wolfe MK, Duong D, Bakker KM, Ammerman M, Mortenson L, Hughes B, et al. Wastewater-Based Detection of Two Influenza Outbreaks. Environ Sci Technol Lett 2022;9:687–92. https://doi.org/10.1021/acs.estlett.2c00350.

[156] Boehm AB, Hughes B, Duong D, Chan-Herur V, Buchman A, Wolfe MK, et al. Wastewater concentrations of human influenza, metapneumovirus, parainfluenza, respiratory syncytial virus, rhinovirus, and seasonal coronavirus nucleic-acids during the COVID-19 pandemic: a surveillance study. Lancet Microbe 2023. https://doi.org/10.1016/S2666-5247(22)00386-X.

[157] Dumke R, Geissler M, Skupin A, Helm B, Mayer R, Schubert S, et al. Simultaneous Detection of SARS-CoV-2 and Influenza Virus in Wastewater of Two Cities in Southeastern Germany, January to May 2022. Int J Environ Res Public Health 2022;19:13374. https://doi.org/10.3390/ijerph192013374.

[158] Mercier E, D'Aoust PM, Thakali O, Hegazy N, Jia J-J, Zhang Z, et al. Municipal and neighbourhood level wastewater surveillance and subtyping of an influenza virus outbreak. Sci Rep 2022;12:15777. https://doi.org/10.1038/s41598-022-20076-z.

[159] Al Khatib HA, Coyle PV, Al Maslamani MA, Al Thani AA, Pathan SA, Yassine HM. Molecular and biological characterization of influenza A viruses isolated from human fecal samples. Infection, Genetics and Evolution 2021;93:104972. https://doi.org/10.1016/j.meegid.2021.104972.

[160] Minodier L, Masse S, Capai L, Blanchon T, Ceccaldi P-E, van der Werf S, et al. Risk factors for seasonal influenza virus detection in stools of patients consulting in general practice for acute respiratory infections in France, 2014-2016. Influenza and Other Respiratory Viruses 2019;13:398–406. https://doi.org/10.1111/irv.12523.

[161] Suess T, Remschmidt C, Schink SB, Schweiger B, Heider A, Milde J, et al. Comparison of Shedding Characteristics of Seasonal Influenza Virus (Sub)Types and Influenza A(H1N1)pdm09; Germany, 2007–2011. PLOS ONE 2012;7:e51653. https://doi.org/10.1371/journal.pone.0051653.

[162] Public Health Agency of Canada. Pathogen Safety Data Sheets: Infectious Substances – Influenza virus type A. Government of Canada 2011.

[163] Public Health Agency of Canada. Pathogen Safety Data Sheets: Infectious Substances – Influenza virus (B and C). Government of Canada 2012.

[164] Santé Publique France. La grippe, une épidémie saisonnière. Santé Publique France n.d.

[165] Khanmohammadi S, Rezaei N. Influenza Viruses. In: Rezaei N, editor. Encyclopedia of Infection and Immunity, Oxford: Elsevier; 2022, p. 67–78. https://doi.org/10.1016/B978-0-12-818731-9.00176-2.

[166] World Health Organization. Influenza (Seasonal) 2023.

[167] Mtetwa HN, Amoah ID, Kumari S, Bux F, Reddy P. Molecular surveillance of tuberculosis-causing mycobacteria in wastewater. Heliyon 2022;8:e08910. https://doi.org/10.1016/j.heliyon.2022.e08910.

[168] Public Health Agency of Canada PHA of. Pathogen Safety Data Sheets: Infectious Substances – Mycobacterium tuberculosis and Mycobacterium tuberculosis complex. Government of Canada 2012.

[169] Churchyard G, Kim P, Shah NS, Rustomjee R, Gandhi N, Mathema B, et al. What We Know About Tuberculosis Transmission: An Overview. J Infect Dis 2017;216:S629–35. https://doi.org/10.1093/infdis/jix362.

[170] World Health Organization. Tuberculosis (TB) 2022.

[171] Institut Pasteur. Tuberculosis. Institut Pasteur 2017.

[172] Santé Publique France. Tuberculose. Santé Publique France 2023.

[173] Santé Publique France. Tuberculose en France : les chiffres 2020. Santé Publique France 2021.

[174] Ministry of Health Ontario. Case Definitions and Disease Specific Information Tuberculosis. Ontario 2022.

[175] Ninove L, Zandotti C, Dambo M, Colson P, Charrel R, Nougairede A. Épidémiologie des infections à coronavirus HCoV-HKU1 à Marseille, France. Médecine et Maladies Infectieuses 2020;50:S195. https://doi.org/10.1016/j.medmal.2020.06.417.

[176] Vabret A, Mourez T, Dina J, van der Hoek L, Gouarin S, Petitjean J, et al. Human coronavirus NL63, France. Emerg Infect Dis 2005;11:1225–9. https://doi.org/10.3201/eid1108.050110.

[177] Liu D, Chen C, Chen D, Zhu A, Li F, Zhuang Z, et al. Mouse models susceptible to HCoV-229E and HCoV-NL63 and cross protection from challenge with SARS-CoV-2. Proceedings of the National Academy of Sciences 2023;120:e2202820120. https://doi.org/10.1073/pnas.2202820120.

[178] Liu DX, Liang JQ, Fung TS. Human Coronavirus-229E, -OC43, -NL63, and -HKU1 (Coronaviridae). Encyclopedia of Virology 2021:428–40. https://doi.org/10.1016/B978-0-12-809633-8.21501-X.

[179] Van Der Hoek L, Pyrc K, Berkhout B. Human coronavirus NL63, a new respiratory virus. FEMS Microbiol Rev 2006;30:760–73. https://doi.org/10.1111/j.1574-6976.2006.00032.x.

[180] Vabret A, Dina J, Gouarin S, Petitjean J, Tripey V, Brouard J, et al. Human (non-severe acute respiratory syndrome) coronavirus infections in hospitalised children in France. Journal of Paediatrics and Child Health 2007;44:176–81. https://doi.org/10.1111/j.1440-1754.2007.01246.x.

[181] Kesheh MM, Hosseini P, Soltani S, Zandi M. An overview on the seven pathogenic human coronaviruses. Reviews in Medical Virology 2022;32:e2282. https://doi.org/10.1002/rmv.2282.

[182] Mulabbi EN, Tweyongyere R, Byarugaba DK. The history of the emergence and transmission of human coronaviruses. Onderstepoort J Vet Res 2021;88:1872. https://doi.org/10.4102/ojvr.v88i1.1872.

[183] Boncristiani HF, Criado MF, Arruda E. Respiratory Viruses. In: Schaechter M, editor. Encyclopedia of Microbiology (Third Edition), Oxford: Academic Press; 2009, p. 500–18. https://doi.org/10.1016/B978-012373944-5.00314-X.

[184] Paloniemi M, Lappalainen S, Vesikari T. Commonly circulating human coronaviruses do not have a significant role in the etiology of gastrointestinal infections in hospitalized children. J Clin Virol 2015;62:114–7. https://doi.org/10.1016/j.jcv.2014.10.017.
[185] Lim YX, Ng YL, Tam JP, Liu DX. Human Coronaviruses: A Review of Virus–Host Interactions. Diseases 2016;4:26. https://doi.org/10.3390/diseases4030026.
[186] Harrison CM, Doster JM, Landwehr EH, Kumar NP, White EJ, Beachboard DC, et al. Evaluating the Virology and Evolution of Seasonal Human Coronaviruses Associated with the Common Cold in the COVID-19 Era. Microorganisms 2023;11:445. https://doi.org/10.3390/microorganisms11020445.
[187] Bibby K, Peccia J. Identification of Viral Pathogen Diversity in Sewage Sludge by Metagenome Analysis. Environ Sci Technol 2013;47:1945–51. https://doi.org/10.1021/es305181x.
[188] Bibby K, Fischer RJ, Casson LW, de Carvalho NA, Haas CN, Munster VJ. Disinfection of Ebola Virus in Sterilized Municipal Wastewater. PLoS Negl Trop Dis 2017;11:e0005299. https://doi.org/10.1371/journal.pntd.0005299.
[189] Centers for Disease Control and Prevention. Signs and Symptoms | Ebola Hemorrhagic Fever | CDC. Centers for Disease Control and Prevention 2022.
[190] European Centre for Disease Prevention and Control. Ebola virus disease. European Centre for Disease Prevention and Control 2010.
[191] Institut Pasteur. Ebola. Institut Pasteur 2021.
[192] National Collaborating Center for Infcetious Diseases. Ebola. National Collaborating Centre for Infectious Diseases 2019.
[193] Public Health Agency of Canada. Ebolaviruses: Infectious substances Pathogen Safety Data Sheet. Government of Canada 2023.
[194] Public Health Agency of Canada. Ebola disease: Symptoms and treatment. Government of Canada 2023.
[195] Schmidt JP, Park AW, Kramer AM, Han BA, Alexander LW, Drake JM. Spatiotemporal Fluctuations and Triggers of Ebola Virus Spillover - Volume 23, Number 3—March 2017 - Emerging Infectious Diseases journal - CDC n.d. https://doi.org/10.3201/eid2303.160101.
[196] UK Health Security Agency. Ebola: overview, history, origins and transmission. GOVUK 2023.
[197] Vetter P, Fischer WA, Schibler M, Jacobs M, Bausch DG, Kaiser L. Ebola virus shedding and transmission: review of current evidence. The Journal of Infectious Diseases 2016;214:S177–84.
[198] World Health Organization. Ebola virus disease. World Health Organization 2021.
[199] World Health Organization. Ebola outbreak in West Africa and the risk to Europe. World Health Organization n.d.
[200] Wang X-W, Li J-S, Guo T-K, Zhen B, Kong Q-X, Yi B, et al. Concentration and detection of SARS coronavirus in sewage from Xiao Tang Shan Hospital and the 309th Hospital. Journal of Virological Methods 2005;128:156–61. https://doi.org/10.1016/j.jviromet.2005.03.022.
[201] Cheng PK, Wong DA, Tong LK, Ip S-M, Lo AC, Lau C-S, et al. Viral shedding patterns of coronavirus in patients with probable severe acute respiratory syndrome. The Lancet 2004;363:1699–700. https://doi.org/10.1016/S0140-6736(04)16255-7.
[202] Public Health Agency of Canada. Pathogen Safety Data Sheets: Infectious Substances – Severe acute respiratory syndrome (SARS) associated coronavirus. Government of Canada 2019.
[203] World Health Organization. Severe Acute Respiratory Syndrome (SARS). World Health Organization n.d.
[204] Centers for Disease Control and Prevention. SARS (10 Years After) | Disease or Condition of the Week | CDC. Centers for Disease Control and Prevention 2016.
[205] Centers for Disease Control and Prevention. SARS | Basics Factsheet | CDC. Centers for Disease Control and Prevention 2017.
[206] HUI DS, WONG P, WANG C. SARS: clinical features and diagnosis. Respirology 2003;8:S20–4. https://doi.org/10.1046/j.1440-1843.2003.00520.x.
[207] World Health Organization. Summary of probable SARS cases with onset of illness from 1 November 2002 to 31 July 2003. World Health Organization 2015.
[208] Girón-Guzmán I, Díaz-Reolid A, Truchado P, Carcereny A, Garcia-Pedemonte D, Hernaez B, et al. Wastewater based epidemiology beyond SARS-CoV-2: Spanish wastewater reveals the current spread of Monkeypox virus 2022:2022.09.19.22280084. https://doi.org/10.1101/2022.09.19.22280084.
[209] Wurtzer S, Levert M, Dhenain E, Boni M, Tournier JN, Londinsky N, et al. First Detection of Monkeypox Virus Genome in Sewersheds in France: The Potential of Wastewater-Based Epidemiology for Monitoring Emerging Disease. Environ Sci Technol Lett 2022;9:991–6. https://doi.org/10.1021/acs.estlett.2c00693.
[210] Wolfe MK, Duong D, Hughes B, Chan-Herur V, White BJ, Boehm AB. Detection of monkeypox viral DNA in a routine wastewater monitoring program 2022:2022.07.25.22278043. https://doi.org/10.1101/2022.07.25.22278043.
[211] Public Health Agency of Canada. Mpox (monkeypox): For health professionals. Government of Canada 2023.
[212] Public Health Agency of Canada. Pathogen Safety Data Sheets: Infectious Substances – Monkeypox virus. Government of Canada 2022.
[213] Centers for Disease Control and Prevention. 2022 Mpox Outbreak Global Map. Centers for Disease Control and Prevention 2023.
[214] Santé Publique France. Monkeypox. Santé Publique France 2023.
[215] Chowdhury PPD, Haque MdA, Ahamed B, Tanbir Md, Islam MdR. A Brief Report on Monkeypox Outbreak 2022: Historical Perspective and Disease Pathogenesis. Clin Pathol 2022;15:2632010X221131660. https://doi.org/10.1177/2632010X221131660.
[216] Centers for Disease Control and Prevention. Animals and COVID-19. Centers for Disease Control and Prevention 2022.
[217] European Centre for Disease Prevention and Control. COVID-19 Infection. European Centre for Disease Prevention and Control 2022.
[218] European Centre for Disease Prevention and Control. Questions and answers on COVID-19: Basic facts. European Centre for Disease Prevention and Control 2022.
[219] Li W-T, Zhang Y, Liu M, Liu Y-Q, Ma X. Prolonged viral shedding in feces of children with COVID-19: a systematic review and synthesis of data. Eur J Pediatr 2022;181:4011–7. https://doi.org/10.1007/s00431-022-04622-5.
[220] Fontana LM, Villamagna AH, Sikka MK, McGregor JC. Understanding viral shedding of severe acute respiratory coronavirus virus 2 (SARS-CoV-2): Review of current literature. Infection Control & Hospital Epidemiology 2021;42:659–68. https://doi.org/10.1017/ice.2020.1273.
[221] Gupta S, Parker J, Smits S, Underwood J, Dolwani S. Persistent viral shedding of SARS-CoV-2 in faeces – a rapid review. Colorectal Disease 2020;22:611–20. https://doi.org/10.1111/codi.15138.
[222] Zhang Y, Cen M, Hu M, Du L, Hu W, Kim JJ, et al. Prevalence and Persistent Shedding of Fecal SARS-CoV-2 RNA in Patients With COVID-19 Infection: A Systematic Review and Meta-analysis. Clinical and Translational Gastroenterology 2021;12:e00343. https://doi.org/10.14309/ctg.0000000000000343.
[223] Miura F, Kitajima M, Omori R. Duration of SARS-CoV-2 viral shedding in faeces as a parameter for wastewater-based epidemiology: Re-analysis of patient data using a shedding dynamics model. Science of The Total Environment 2021;769:144549. https://doi.org/10.1016/j.scitotenv.2020.144549.
[224] UK Health Security Agency. COVID-19: epidemiology, virology and clinical features. GOVUK 2022.
[225] D'Amico F, Marmiere M, Righetti B, Scquizzato T, Zangrillo A, Puglisi R, et al. COVID-19 seasonality in temperate countries. Environ Res 2022;206:112614. https://doi.org/10.1016/j.envres.2021.112614.
[226] Jones PW, Rennison LM, Matthews PR, Collins P, Brown A. The occurrence and significance to animal health of Leptospira, Mycobacterium, Escherichia coli, Brucella abortus and Bacillus anthracis in sewage and sewage sludges. J Hyg (Lond) 1981;86:129–37. https://doi.org/10.1017/s0022172400068820.
[227] Institut Pasteur. Leptospirosis. Institut Pasteur 2020.
[228] Public Health Agency of Canada. Pathogen Safety Data Sheets: Infectious Substances – Leptospira interrogans. Government of Canada 2011.
[229] Bourhy P, Septfons A, Picardeau M. Diagnostic, surveillance et épidémiologie de la leptospirose en France. Bull Epidemiol Hebd 2017;8:131–7.
[230] Picardeau M. Rapports d'activité du CNR de la Leptospirose 2020-2021. 2021.
[231] Lass A, Ma L, Kontogeorgos I, Xueyong Z, Li X, Karanis P. Contamination of wastewater with Echinococcus multilocularis – possible implications for drinking water resources in the QTP China. Water Research 2020;170:115334. https://doi.org/10.1016/j.watres.2019.115334.
[232] Public Health Agency of Canada. Pathogen Safety Data Sheets: Infectious Substances – Echinococcus multilocularis. Government of Canada 2015.
[233] Ministère de l'Agriculture et de la Souveraineté alimentaire. L'échinococcose alvéolaire. Ministère de l'Agriculture et de la Souveraineté alimentaire 2017.
[234] Piarroux M, Bresson-Hadni S, Capek I, Knapp J, Watelet J, Dumortier J, et al. Surveillance de l'échinococcose alvéolaire en France : bilan de cinq années d'enregistrement, 2001-2005. Numéro thématique. Les zoonoses en France 2006.
[235] Prevention C-C for DC and. CDC - Echinococcosis - Biology. Centers for Disease Control and Prevention 2019.
[236] Terwilliger A, Clark J, Avadhanula V, Feliz K, Weesner K, Murphy E, et al. HIV Detection in Wastewater as a Potential Epidemiological Bellwether 2022. https://doi.org/10.2139/ssrn.4257909.
[237] HIV and AIDS. World Health Organization 2023.
[238] Public Health Agency of Canada. Pathogen Safety Data Sheets: Infectious Substances – Human immunodeficiency virus (HIV). Government of Canada 2011.
[239] Xu B, Li J, Wang M. Epidemiological and time series analysis on the incidence and death of AIDS and HIV in China. BMC Public Health 2020;20:1906. https://doi.org/10.1186/s12889-020-09977-8.
[240] World Health Organization. HIV n.d.
[241] ANRS. Priorities - HIV/AIDS - SHS | ANRS n.d.
[242] UNAIDS. UNAIDS Data France n.d.
[243] Zhou N, Ong A, Fagnant-Sperati C, Harrison J, Kossik A, Beck N, et al. Evaluation of Sampling and Concentration Methods for Salmonella enterica Serovar Typhi Detection from Wastewater. The American Journal of Tropical Medicine and Hygiene 2023;108:482–91. https://doi.org/10.4269/ajtmh.22-0427.
[244] World Health Organization. Typhoid. World Health Organization 2018.
[245] Centers for Disease Control and Prevention. About Global Typhoid Fever. Centers for Disease Control and Prevention 2023.
[246] European Centre for Disease Prevention and Control. Typhoid and paratyphoid fevers: Annual Epidemiological Report for 2017 2020.
[247] Colomba C, Saporito L, Titone L. Typhoid Fever. In: Heggenhougen HK (Kris), editor. International Encyclopedia of Public Health, Oxford: Academic Press; 2008, p. 414–20. https://doi.org/10.1016/B978-012373960-5.00619-5.
[248] Institut Pasteur. Typhoid and paratyphoid fever. Institut Pasteur 2021.
[249] Svraka S, Vennema H, van der Veer B, Hedlund K-O, Thorhagen M, Siebenga J, et al. Epidemiology and Genotype Analysis of Emerging Sapovirus-Associated Infections across Europe. Journal of Clinical Microbiology 2010;48:2191–8. https://doi.org/10.1128/JCM.02427-09.
[250] Oka T, Wang Q, Katayama K, Saif LJ. Comprehensive Review of Human Sapoviruses. Clin Microbiol Rev 2015;28:32–53. https://doi.org/10.1128/CMR.00011-14.
[251] Zhuo R, Ding X, Freedman SB, Lee BE, Ali S, Luong J, et al. Molecular Epidemiology of Human Sapovirus among Children with Acute Gastroenteritis in Western Canada. J Clin Microbiol n.d.;59:e00986-21. https://doi.org/10.1128/JCM.00986-21.
[252] Haut Conseil de la Santé Publique. Recommandations relatives aux conduites à tenir devant des gastro-entérites aiguës en établissement d'hébergement pour personnes âgées 2010.
[253] Centers for Disease Control and Prevention. About MERS. Centers for Disease Control and Prevention 2019.
[254] Cevik M, Tate M, Lloyd O, Maraolo AE, Schafers J, Ho A. SARS-CoV-2, SARS-CoV-1 and MERS-CoV viral load dynamics, duration of viral shedding and infectiousness – a living systematic review and meta-analysis 2020:2020.07.25.20162107. https://doi.org/10.1101/2020.07.25.20162107.

[255] European Centre for Disease Prevention and Control. Factsheet about Middle East respiratory syndrome coronavirus (MERS-CoV). European Centre for Disease Prevention and Control 2017.
[256] European Centre for Disease Prevention and Control. MERS-CoV worldwide overview. European Centre for Disease Prevention and Control 2023.
[257] Government of South Australia A. Middle East respiratory syndrome (MERS) - including symptoms, treatment and prevention. Government of South Australia n.d.
[258] Institut Pasteur. MERS-CoV. Institut Pasteur 2015.
[259] Public Health Agency of Canada. Pathogen Safety Data Sheet: Infectious substances - Middle east respiratory syndrome (MERS)-related Coronavirus. Government of Canada 2019.
[260] World Health Organization. Middle East respiratory syndrome coronavirus (MERS-CoV). World Health Organization n.d.
[261] Corman VM, Albarrak AM, Omrani AS, Albarrak MM, Farah ME, Almasri M, et al. Viral Shedding and Antibody Response in 37 Patients With Middle East Respiratory Syndrome Coronavirus Infection. Clinical Infectious Diseases 2016;62:477–83. https://doi.org/10.1093/cid/civ951.
[262] Drosten C, Seilmaier M, Corman VM, Hartmann W, Scheible G, Sack S, et al. Clinical features and virological analysis of a case of Middle East respiratory syndrome coronavirus infection. The Lancet Infectious Diseases 2013;13:745–51. https://doi.org/10.1016/S1473-3099(13)70154-3.
[263] Zumla A, Hui DS, Perlman S. Middle East respiratory syndrome. The Lancet 2015;386:995–1007. https://doi.org/10.1016/S0140-6736(15)60454-8.
[264] World Health Organization. Middle East respiratory syndrome coronavirus (MERS-CoV). World Health Organization 2022.
[265] INRS. Hépatite B. INRS 2018.
[266] Bouvet de la Maisonneuve P, Legris C, De Saunière A, Dufay B. Bulletin de veille sanitaire - N°28 spécial Hépatites B et C/ 20 juin 2017. Région Ile-de-France: Santé Publique France; 2017.
[267] Institut Pasteur. Viral hepatitis. Institut Pasteur 2021.

**Supplementary Table S2**: Summary of consensus reached per pathogen for the three rounds of the Delphi survey

Five pathogen categories that respected the analytical feasibility criterion were suggested for evaluation after the first Delphi round. These are marked as "pathogen not evaluated" for round 1 in the table below.

| **Potential wastewater surveillance targets for the Paris 2024 OPG** | | **Delphi survey iteration** | | |
|---|---|---|---|---|
| Disease | Pathogen species /genogroup | Round 1 | Round 2 | Round 3 |
| Adenovirus | Human adenovirus-F | | 85%; 28/33 | |
| *Alphacoronavirus* | *HCoV-229E* | | 82%; 27/33 | |
| Amoebiasis | *Entamoeba histolytica* | 76%; 25/33 | | |
| *Betacoronavirus* | *Betacoronavirus 1, HCoV-HKU1* | | 82%; 27/33 | |
| Campylobacter infections | 17 species have been identified, of which C. jejuni and C. coli are most frequently reported in human disease | 82%; 27/33 | | |
| Cholera | *Vibrio cholerae* | | | |
| Coronavirus disease | *SARS-CoV-2* | 91%; 30/33 | | |
| *Cryptosporidium* | *C. hominis, C. parvum, C. meleagridis, C. muris* | | 73%; 24/33 | |
| Ebola | Zaire ebolavirus, Sudan ebolavirus, Tai Forest ebolavirus, Bundibugyo ebolavirus | | | 78%; 25/32 |
| Echinococcosis | *Echinococcus multilocularis* | 91%; 30/33 | | |
| *Escherichia coli infections* | *Escherichia coli* | | 88%; 29/33 | |
| *Giardia* | *G. duodenalis* | | 85%; 29/33 | |
| Hepatitis A | Hepatitis A virus | | | |
| Hepatitis E | Hepatitis E virus | | 85%; 28/33 | |
| Human immunodeficiency virus (HIV) infection | Human immunodeficiency virus 1, Human immunodeficiency virus 2 | 91%; 30/33 | | |
| *Influenza* | Influenza A virus | | 70%; 23/33 | |
| | Influenza B virus | | 70%; 23/33 | |
| Legionnaires' disease | *20 species have been documented as human pathogens, of which L. pneumophila most commonly causes of illness humans* | 76%; 25/33 | | |
| Leptospirosis | 64 species have been identified | 73%; 24/33 | | |
| Measles | Measles virus (MV) | | | |
| Mpox | Mpox virus | 85%; 28/33 | | |
| Non-polio enterovirus | Enterovirus A, B, C and D | | | 78%; 25/32 |
| *Norovirus* | GI, GII and GIV | | | |
| Poliomyelitis | Poliovirus | 85%; 28/33 | | |
| *Rotavirus* | Rotavirus A | | 73%; 24/33 | |
| Salmonellosis (Nontyphoidal Salmonella) | *Salmonella bongori, Salmonella enterica* | | 88%; 29/33 | |
| Severe acute respiratory syndrome (SARS) | *SARS-CoV-1* | | | 78%; 25/32 |
| Shigellosis | *Shigella sonnei, Shigella flexneri, Shigella boydii, Shigella dysenteriae* | | | |
| Tuberculosis | Mycobacterium tuberculosis complex | 70%; 23/33 | | |
| Yersiniosis | *Yersinia enterocolitica* | 82%; 27/33 | | |

**Legend**

- % Consensus reached for inclusion
- % Consensus reached for exclusion
- No consensus reached
- Pathogen not evaluated

**Supplementary Figure S1:** Expert opinion on the most relevant prospective pathogens for wastewater-related research (development of detection methods, etc.) amongst the 29 excluded on the basis of dissatisfaction to the analytical feasibility criterion

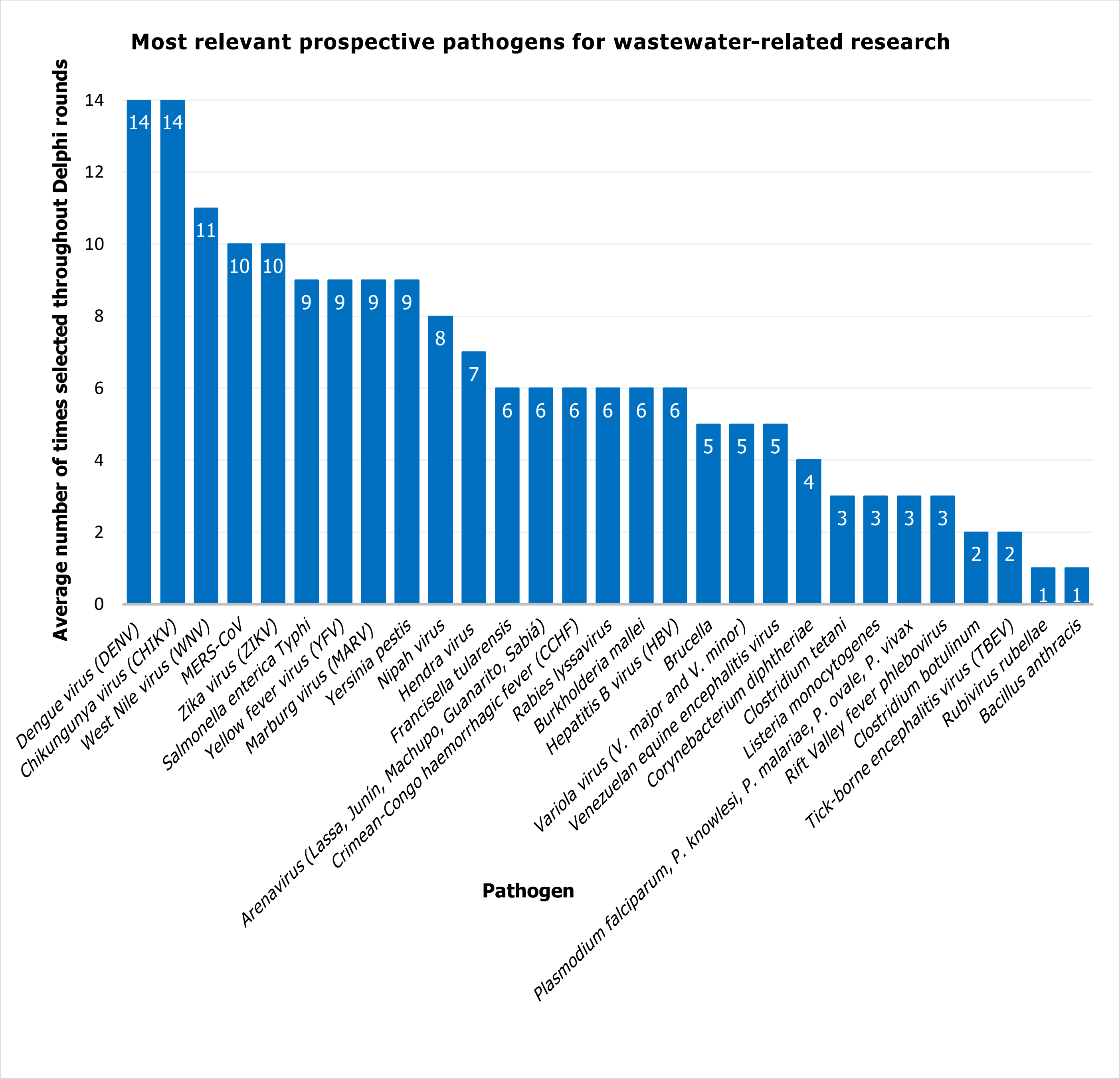

Supplement: Supplement [file 24-00231_TORO_Supplement.pdf]
